# Tumor marker–guided precision BNCT for CA19-9–positive cancers: a new paradigm in molecularly targeted chemoradiation therapy

**DOI:** 10.1186/s12967-025-07349-7

**Published:** 2025-12-08

**Authors:** Noriyuki Kanehira, Fuminori Teraishi, Tomoyuki Tajima, Tatsunori Osone, Kazuyoshi Gotoh, Takuya Fujimoto, Yoshinori Sakurai, Natsuko Kondo, Narikazu Nagahisa, Kaoru Kamei, Taiga Fujita, Akira Morihara, Yutaka Takaguchi, Mizuki Kitamatsu, Takeshi Takarada, Kunitoshi Shigeyasu, Minoru Suzuki, Toshiyoshi Fujiwara, Hiroyuki Michiue

**Affiliations:** 1 Department of Gastroenterological Surgery, Okayama University Graduate School of Medicine, Dentistry and Pharmaceutical Sciences, 2-5-1 Shikata-Cho, Kita-Ku, Okayama City, Okayama, 700-8558 Japan; 2 Neutron Therapy Research Center, Okayama University, 2-5-1 Shikata-Cho, Kita-Ku, Okayama City, Okayama, 700-8558 Japan; 3 Graduate School of Environmental, Life, Natural Science and Technology, Okayama University, 3-1-1 Tsushima-Naka, Kita-ku, Okayama, 700-8530 Japan; 4 Department of Regenerative Science, Okayama University Graduate School of Medicine, Dentistry and Pharmaceutical Sciences, 2-5-1 Shikata-Cho, Kita-Ku, Okayama City, Okayama, 700-8558 Japan; 5 Department of Medical Laboratory Science, Okayama University Graduate School of Health Sciences, 2-5-1 Shikata-Cho, Kita-Ku, Okayama City, Okayama, 700-8558 Japan; 6 Institute for Integrated Radiation and Nuclear Science, Kyoto University, Osaka, Japan; 7 Graduate School of Environmental, Life Science, Okayama University, 3-1-1, Tsushima-Naka, Kita-Ku, Okayama, 700-8530 Japan; 8 Faculty of Sustainable Design, Department of Material Design and Engineering, University of Toyama, Gofuku, Toyama, 3190 Japan; 9 Department of Applied Chemistry, Kindai University, 3-4-1 Kowakae, Osaka, Higashi-Osaka 577-8502 Japan

**Keywords:** Boron neutron capture therapy (BNCT), Precision BNCT, Fucose-conjugated medicine, CA19-9, Drug discovery

## Abstract

**Background:**

Boron neutron capture therapy (BNCT) is a molecularly targeted chemoradiation modality that relies on boron delivery agents such as p-borophenylalanine (BPA), which require LAT1 (L-type amino acid transporter 1) for tumor uptake. However, the limited efficacy of BPA in LAT1-low tumors restricts its therapeutic scope. To address this limitation, we developed a tumor marker–guided BNCT strategy targeting cancers overexpressing the clinically validated glycan biomarker CA19-9.

**Methods:**

We conducted transcriptomic analyses using The Cancer Genome Atlas (TCGA) datasets to identify LAT1-low cancers with high CA19-9 expression. These analyses revealed elevated expression of fucosyltransferase 3 (FUT3), which underlies CA19-9 biosynthesis, in pancreatic, biliary, and ovarian malignancies. Based on this, we synthesized a novel boron compound, fucose-BSH, designed to selectively accumulate in CA19-9–positive tumors. We evaluated its physicochemical properties, pharmacokinetics, biodistribution, and antitumor efficacy in cell lines and xenograft models, comparing its performance to that of BPA.

**Results:**

Fucose-BSH demonstrated significantly greater boron uptake in CA19-9–positive cell lines (AsPC-1, Panc 04.03, HuCCT-1, HSKTC, OVISE) compared to CA19-9–negative PANC-1. In HuCCT-1 xenografts, boron accumulation reached 36.2 ppm with a tumor/normal tissue ratio of 2.1, outperforming BPA. Upon neutron irradiation, fucose-BSH–mediated BNCT achieved > 80% tumor growth inhibition. Notably, fucose-BSH retained therapeutic efficacy in LAT1-deficient models where BPA was ineffective, confirming LAT1-independent targeting.

**Conclusions:**

This study establishes a novel precision BNCT approach by leveraging CA19-9 as a tumor-selective glycan marker for boron delivery. Fucose-BSH offers a promising platform for expanding BNCT to previously inaccessible LAT1-low malignancies, including pancreatic, biliary, and ovarian cancers. These findings provide a clinically actionable strategy for tumor marker–driven chemoradiation and lay the foundation for translational application in BNCT. This strategy has the potential to support companion diagnostic development and precision stratification in ongoing and future BNCT clinical trials.

**Translational Relevance:**

Malignancies with elevated CA19-9 expression, such as pancreatic, biliary, and ovarian cancers, are associated with poor prognosis and limited response to current therapies. This study presents a tumor marker–guided strategy for boron neutron capture therapy (BNCT) by leveraging CA19-9 glycan biology to enable selective tumor targeting via fucose-BSH, a novel boron compound. Through transcriptomic data mining and preclinical validation, fucose-BSH demonstrated LAT1-independent boron delivery, potent BNCT-mediated cytotoxicity, and tumor-specific accumulation in CA19-9–positive models. These findings support a precision chemoradiation approach that addresses a critical gap in BNCT applicability, offering a clinically actionable pathway for patient stratification and therapeutic development in CA19-9–expressing cancers.

**Graphical Abstract:**

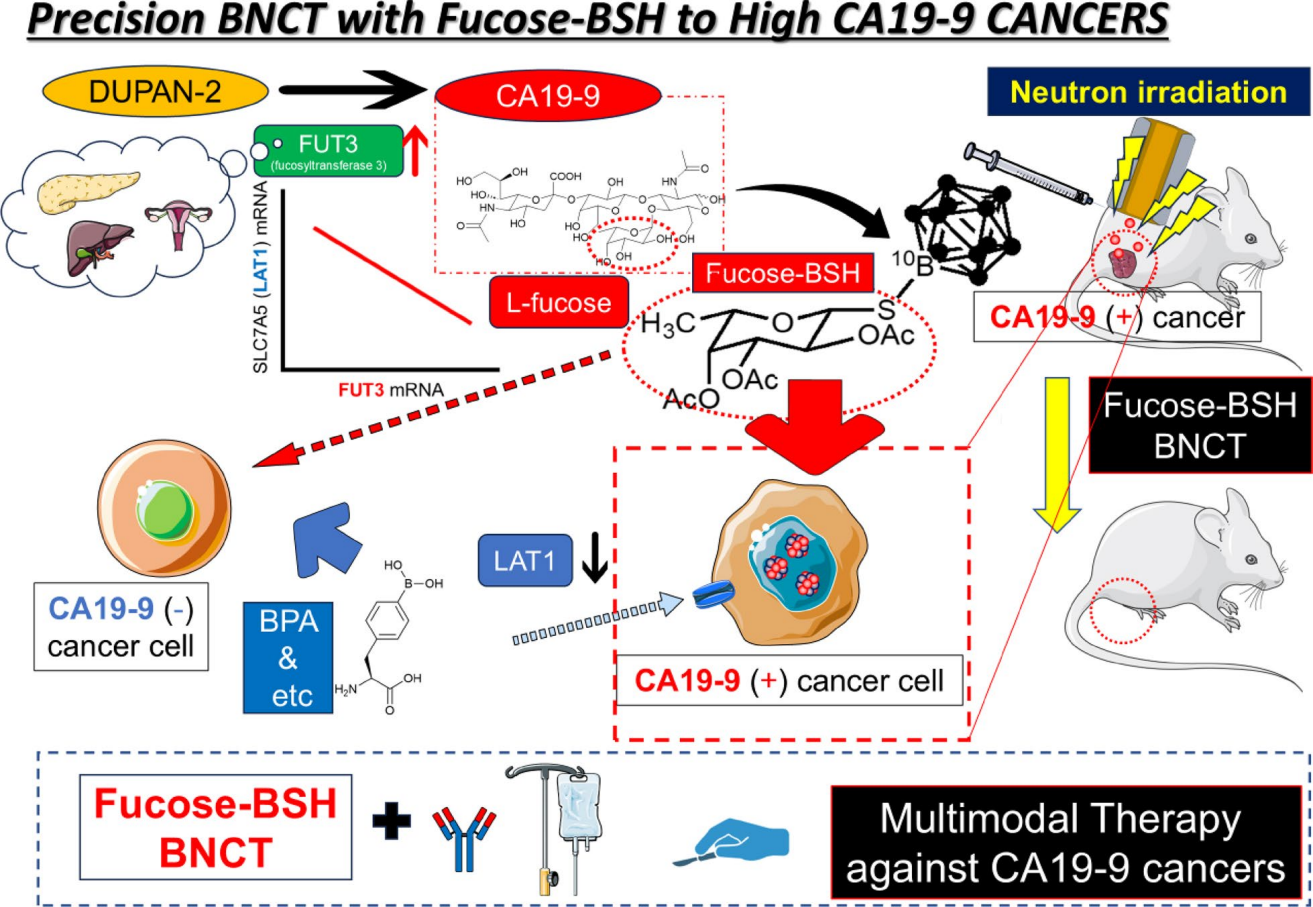

**Supplementary information:**

The online version contains supplementary material available at 10.1186/s12967-025-07349-7.

## Introduction

Boron neutron capture therapy (BNCT) was proposed by Dr. Gordon Locher in 1936 as a treatment method in which a boron atom (10 B)-containing drug is administered to a cancer patient, while the same site is simultaneously irradiated with neutrons. The drug is highly concentrated, which results in a cell-killing effect through the induced nuclear reaction between neutrons and boron [1, 2]. The range of He particles and Li nuclei produced by the boron-neutron capture reaction is within 10 µm, allowing the specific introduction of 10B-containing drugs into target cancer cells to enable treatment at the cellular level without affecting the surrounding normal cells [3]. Because BNCT is a chemoradiotherapy at the cellular level, the key to successful treatment is the specific introduction of boron drugs into cells. The first BNCT for recurrent head and neck cancer was approved by Japanese public medical insurance [4]. Drugs currently used in BNCT include p-boronophenylalanine (BPA) and sodium borocaptate (BSH) [5]. One approved boron preparation method is the preparation of boron amino acid (BPA) with phenylalanine [4]. As phenylalanine is a substrate for melanin synthesis, a BPA-BNCT clinical trial was conducted by Hatanaka et al. against malignant melanoma with increased melanin biosynthesis, and its therapeutic effect was demonstrated [6, 7]. Subsequently, it was reported that BPA can be introduced into cells not only by melanoma but also by L-amino acid transporters (LAT1) that are highly expressed in various malignant tumors. High uptake has been confirmed in head and neck cancers, malignant brain tumors, and skin malignancies [8]. The development of therapeutic agents other than BPA is also urgently needed because BNCT is highly dependent on the uptake of boron drugs into malignant tumors for its therapeutic efficacy [9]. BSH, which has been used in clinical research for a long time, has not been used in recent years because of its lack of tumor selectivity and its inability to be introduced into cells. Therefore, we came up with the idea of performing a new precision BNCT using BSH with tumor markers because it targets a different target from BPA.

Tumor markers are biological indicators of the presence of tumors [10]. In clinical practice, these molecules can be detected in plasma and other body fluids, and are measurable biochemicals associated with malignant tumors [11]. These markers are produced either directly by tumor cells (tumor-derived) or by the body in response to tumor cells (tumor-related) [12]. Tumor markers include a variety of substances such as cell surface antigens, cytoplasmic proteins, enzymes, hormones, oncofetal antigens, receptors, oncogenes, and their products [13, 14]. One representative tumor marker is carbohydrate antigen 19–9 (CA19-9), also known as sialyl-Lewis A (sLea). CA19-9 is a cell surface glycoprotein complex that was first reported in 1979 in a colon cancer cell line (SW1116) using a mouse monoclonal antibody (1116-NS-19–9) [15, 16]. In 1982, monosialoganglioside was determined to be a cell membrane glycolipid-derived sugar chain recognized by this antigen, particularly for the diagnosis and treatment of pancreatic cancer. In 1979, Koprowski et al. discovered a specific antigen in the blood of patients with pancreatic cancer and named it CA19-9, reporting it to be a pancreatic cancer-specific antigen [16]. In the 1980s, CA19-9 levels were elevated not only in pancreatic cancer, but also in other digestive cancers, such as biliary tract cancer, stomach cancer, and colon cancer. Serum, saliva, pancreatic cysts, ascites, pleural fluid, and bronchoalveolar lavage (BAL) fluid were used to examine CA19-9 levels [15]. This antigen is now used in combination with various imaging, hematological, pathological, and genetic diagnostic techniques and other clinical evaluations.CA19-9 is formed by the biosynthesis of fucose and glucose taken up by cells as GDP-L-fucose into the Golgi apparatus and DUPAN-2 via FUT3. Elevated urinary fucose levels have been reported as an early pancreatic cancer diagnostic biomarker, with two related markers, blood CA19-9 and urinary fucose, also being of interest [17]. Recently, CA19-9 is not only an early diagnostic biomarker, but is now also used as a biomarker in various applications before, during, and after treatment [18]. Surgery is generally avoided for localized pancreatic cancer, even for operable lesions, if neoadjuvant chemotherapy (NAC) is performed and CA19-9 levels are high, because the post-surgery prognosis is poor [19]. Furthermore, prognosis following treatment has been reported according to changes in post-operative CA19-9 levels [20]. Even after treatment, it is important to periodically examine CA19-9 levels in combination with imaging to evaluate for potential recurrence. CA19-9 has also become a potential treatment target rather than a biomarker for cancer progression [21]. CA19-9 itself can bind to the extracellular matrix, activate the epidermal growth factor receptor (EGFR) signaling pathway, and interact with the KrasG12D oncogene to promote pancreatic cancer growth [22, 23]. CA19-9 has also been shown to bind to E-secretin on the surface of peritoneal endothelial and mesothelial cells to support metastasis [24]. Therefore, therapeutic antibodies targeting CA19-9, CA19-9 antibody imaging reagents, and CA19-9 antibody nuclear medicine therapies are currently being actively used, with CA19-9-related malignancies attracting substantial attention [21, 25–27].

We propose the development of a novel BSH-based boron-containing drug by chemically conjugating the main component of the CA19-9 skeleton with BSH. These CA19-9-targeted boron-containing drugs represent a novel BNCT strategy targeting pancreatic, bile duct, and related cancers with elevated CA19-9 levels. This strategy aims to achieve tumor-selective boron accumulation, expand the indications for BNCT, and ultimately improve the treatment indices and clinical outcomes in patients with CA19-9-expressing cancers.

## Materials and methods

### Ethics statement

Animal use and care procedures complied with ARRIVE 2.0, and were approved by Okayama University (OKU-2020706, 2021515) and Kyoto University Institute for Integrated Radiation and Nuclear Science (KURNS) ethics committee (2023–38). The mice were housed in Clare Japan plastic cages on AP Anicon racks (Seiken Corp.) at 20–25 °C, 40%-60% humidity, ≥50 air changes/h, and a 12-hour light/dark cycle (lights on at 8:00). The cage density followed standards. Bedding (Eco Chip CL-4163, CLEA Japan) was provided and the mice were fed an MF diet (Oriental Yeast Co.) and sterile water ad libitum. All materials, including the individually ventilated cages, lids, feeders, bottles, and bedding, were autoclaved. The mice were confirmed to be negative for murine infectious agents. The use of clinical samples at Okayama University Hospital was approved by the Ethics Committee (OKU-1903–037), and patient consent was obtained.

### Data source and processing

Gene expression and clinical data from 13 tumors were obtained from The Cancer Genome Atlas (TCGA) database (https://portal.gdc.cancer.gov/). SLC7A5 and FUT3 were analyzed using the cBio Cancer Genomics Portal (cBioPortal). The gene expression profiles of tumor cell lines involved in CA19-9 synthesis were obtained from CCLE (Cancer Cell Line Encyclopedia) (https://sites.broadinstitute.org/ccle/). A protein similarity search was performed using the Basic Local Alignment Search Tool (BLAST) and the non-redundant protein sequence database (nr) with 90% query cover. The EggNOG v5.0. database (http://eggnog5.embl.de/) was used to visualize the evolutionary history of the FucP protein, with a 90% of Query cover threshold. Kaplan-Meier survival analysis was performed using Kaplan-Meier Plotter (https://kmplot.com/analysis/).

### Synthesis of (fucose triacetate)-^**10**^**BSH conjugate (2)**

An acetonitrile (6.7 mL) solution of 2,3,4-tri-O-acetyl-6-deoxy-*α*–L-galactopyranosyl bromide (36 mg, 0.10 mmol), triethylamine (0.4 mL, 3.0 mmol), and Na_2_[^10^B_12_H_11_SH] (64 mg, 0.31 mmol) **1** was stirred at room temperature for 3 days under an Ar atmosphere. After stirring for 3 d, the insoluble solids were collected by filtration. Washing with a small amount of water afforded 1,2,3,4,5,6,7,8,9,10,11-undecahydro-12-(1-thio-*β*-L-fucopyranose 2,3,4-triacetato-S)-[^10^B]dodecaborate **2** [7.4 mg, 15 μmol, and 15% yield (^10^B content: 99%)]. **2**: ^1^H NMR (600 MHz, D_2_O) δ 1.01–1.62 (m, 11 H), 1.16 (d, *J* = 6.6 Hz, 3 H), 1.99 (s, 3 H), 2.12 (s, 3 H), 2.21 (s, 3 H), 4.01 (q, *J* = 6.6 Hz, 1 H), 4.95 (t, *J* = 9.9 Hz, 1 H), 5.16 (dd, *J* = 9.9 and 3.0 Hz, 1 H), 5.28 (d, *J* = 3.0 Hz, 1 H); ^11^B NMR (192 MHz, D_2_O) δ −20.4, −16.9, −14.8, −10.4; ^13^C NMR (150 MHz, D_2_O) δ 16.0, 20.6, 20.7, 21.3, 71.2, 72.0, 73.3, 73.4, 86.5, 173.3, 174.0, 174.3; FT-IR (KBr): 2483, 1734, 1418, 1075, 1002, 736 cm^−1^; ESI-TOF (*m/z*): calcd for C_12_H_28_^10^B_12_NaO_7_S, 459.3011 [M + Na]^–^; found 459.2998 [M + Na]^–^.

### Cell lines and cell culture

Human pancreatic cancer cell lines (PANC-1, AsPC-1, Panc 04.03, and PK-45 H), cholangiocarcinoma (HuCCT-1), Krukenberg tumor (HSKTC), and ovarian cancer (OVISE) cell lines were obtained from ATCC, RIKEN BRC, or JCRB Cell Bank and cultured according to their protocols.

### X-ray irradiation of cells and colony formation assay

PANC-1, PK-45 H, HuCCT-1, OVISE, HSKTC, and Panc 04.03 cells were irradiated with 0–6 Gy X-rays (MX-80Labo) and subcultured for 14 days. Colonies were stained with crystal violet and analyzed using ImageJ software [28].

### Immunocytochemistry (ICC) analysis of human CA19-9-high cells

ICC was performed to assess the BSH distribution using an anti-BSH mAb (a gift from Prof. M. Kirihata, Osaka Prefectural University). Cells incubated with fucose-BSH (2, 6, or 12 h) were fixed, stained with anti-BSH mAb and Alexa Fluor 594, and counterstained with Hoechst 33,258 and phalloidin 488. Fluorescence was observed using a confocal microscope (LSM780, Carl Zeiss, Germany).

### Inductively coupled plasma (ICP) measurement of intracellular boron uptake

All cell samples were analyzed by ICP-MS (Agilent 7900 ICP-MS, Agilent Technologies Inc., Santa Clara, CA, USA) using a previously described method [29]. The ICP was measured at the Okayama University Institute of Plant Science and Resources.

### Inhibition of fucose-BSH uptake

The inhibition of drug uptake was tested as previously described [29]. Cells were cultured in 0.1–10 mM L-fucose media, and viability was assessed at 24 and 48 h by measuring the absorbance at 450 nm. Boron uptake was measured by ICP after 48 h. The GLUT1 inhibitor WZB117 (1–10 µM) was added 24 h later, and boron levels were analyzed 24 h later.

### Pharmacokinetic evaluation of boron drugs in tumor models

All procedures were approved by Okayama University (OKU-2020706, OKU-2021515). Female BALB/c-nude mice (6–8 weeks, 16–20 g, Japan CLEA, Inc.) were inoculated with HuCCT-1 cells (2 × 10^6^ cells/mouse), and fucose-BSH (100 mg/kg) was administered i.p. one week later. Mice were sacrificed at 0.5–4 h, and boron levels in tumors and organs were measured using ICP as previously described [29].

#### *In vitro* and in vivo neutron irradiation experiments

The cells cultured at Okayama University were transported to KURNS and irradiated 24 h later. HuCCT-1 and PANC-1 cells were pre-treated with 150 µM fucose-Ac-BSH or BPA. Irradiation was performed at 1 MW using a mixed neutron mode at the KUR-HWNIF (Heavy Water Neutron Irradiation Facility of Kyoto University Reactor). Cells were then cultured for two weeks, and colony formation was assessed (*n* = 4) using crystal violet staining and ImageJ analysis [28].

BALB/c-nu/nu mice (4 weeks old, female) were subcutaneously injected with HuCCT-1 cells (2 × 10^6^/mouse). Two weeks later, the mice were transported to KURNS and anesthetized (medetomidine/midazolam/butorphanol) before the administration of BPA or fucose-BSH (100 mg/kg). Irradiation was performed at KUR-HWNIF (5 MW, 12 min) with LiF shielding, excluding the tumor. The tumor growth and weight were monitored. All procedures were approved by Okayama University (OKU-2020706, 2021515) and Kyoto University (KURNS 2023–38).

### RNA-seq analysis and statistical analysis

RNA-seq was performed on pancreatic cancer samples from the Okayama University Hospital. The reads were trimmed using Fastp and pseudoaligned to GRCh38 using Kallisto. GeTMM normalization (edgeR) and DEG analysis (NOISeq; prob > 0.8, |Log2FC| > 1) were performed. Data are available from GEO (GSE214899). Statistical tests (t-test, Mann-Whitney U, one-/two-way ANOVA) were performed using GraphPad Prism 9, with significance set at *p* < 0.05 and q < 0.1.

## Results

### Bioinformatics analysis of the CA19-9 synthesis pathway in CA19-9-high and CA19-9-normal cancers

**Fig. 1:**
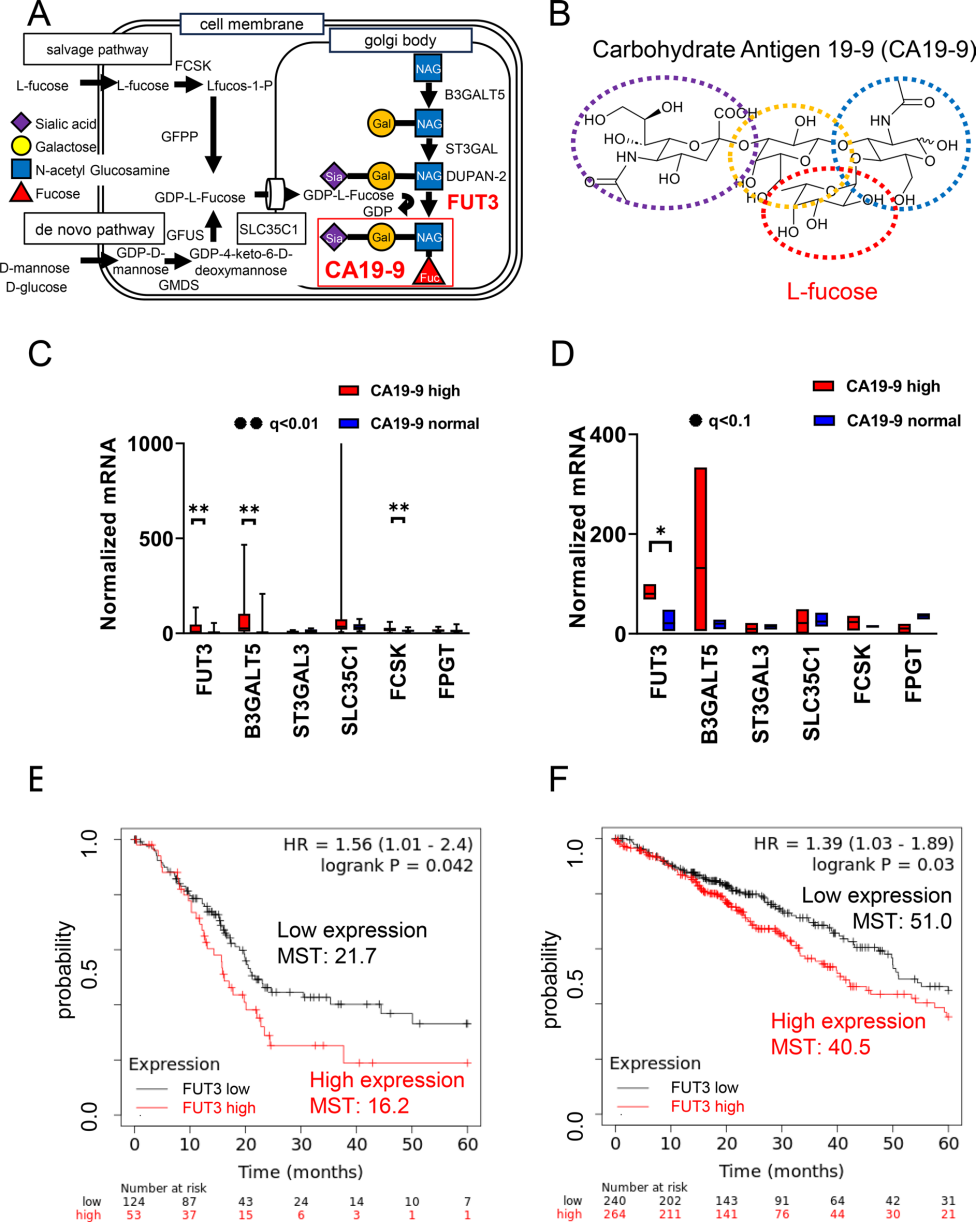
Biosynthesis pathway of carbohydrate antigen 19–9 (CA19-9) and clinical relevance of fucosyltransferase expression. (**A**) schematic illustration of the CA19-9 biosynthetic pathway. (**B**) chemical structures of CA19-9 and its monosaccharide component, L-fucose. (**C**) comparative transcriptomic analysis using the cancer cell Line encyclopedia (ccle): gene expression profiles in CA19-9–high versus CA19-9–normal cell lines (q < 0.01). (**D**) rna sequencing of pancreatic tumor samples comparing CA19-9–high (poor prognosis) and CA19-9–low (favorable prognosis) cases. (**E**) Kaplan–Meier survival analysis of pancreatic ductal adenocarcinoma (pdac) patients stratified by FUT3 expression. (**F**) Kaplan–Meier survival analysis of lung adenocarcinoma patients stratified by FUT3 expression

CA19-9 is synthesized in the Golgi apparatus by galactosyltransferase (B3GALT5), sialyltransferase (ST3GAL3), and fucosyltransferase (FUT3) on N-acetylglucosamine (Fig. 1A). The structure shows N-acetylglucosamine (blue dots), galactose (yellow dots), sialic acid (purple dots), and L-fucose (red dots) (Fig. 1B).

CCLE data showed that CA19-9-high cancer cell lines had significantly upregulated FUT3 (q = 0.003), B3GALT5 (q = 0.003), and FCSK (q = 0.003) mRNA levels compared with CA19-9-normal cell lines (Fig. 1C). RNA-seq of pancreatic cancer samples revealed that FUT3 expression was higher in the poor prognosis group with elevated CA19-9 levels (q = 0.062) (Fig. 1D; Supplementary Figure S1).

Kaplan-Meier analysis indicates shorter median survival in FUT3-high pancreatic ductal adenocarcinoma (PDAC) (16.2 months) versus FUT3-low groups (21.7 months, HR = 1.56, *p* = 0.042) (Fig. 1E), and in lung adenocarcinoma (40.5 versus 51.0 months, HR = 1.39, *p* = 0.03) (Fig. 1F).

CA19-9 pathway bioinformatics: CA19-9 synthesis relies on FUT3/B3GALT5; FUT3 is elevated in CA19-9-high cancers and associates with poorer prognosis in PDAC and lung adenocarcinoma.

### Bioinformatics analysis of amino acid transporter (LAT1) and fucosyltransferase (FUT3) using TCGA

**Fig. 2:**
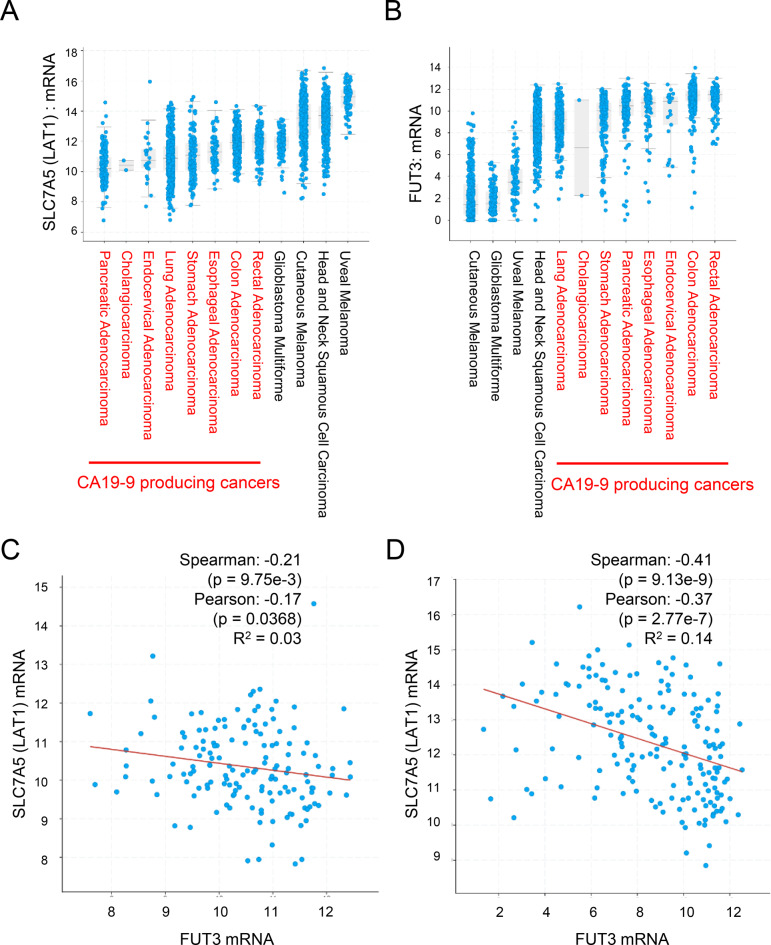
Expression profiles and correlation analysis of LAT1 and FUT3 in CA19-9–positive cancers. (**A**) LAT1 expression in CA19-9–high tumors from the cancer genome atlas (TCGA) dataset. (**B**) FUT3 expression in the same cohort. (**C**) inverse correlation between LAT1 and FUT3 expression in pdac. (**D**) inverse correlation between LAT1 and FUT3 in esophageal adenocarcinoma. All gene expression data were obtained from TCGA RNA-seq datasets and analyzed using Spearman’s correlation

LAT1 and FUT3 were analyzed using TCGA datasets. CA19-9-high tumors exhibited low SLC7A5 (LAT1) expression (Fig. 2A) and high FUT3 expression (Fig. 2B). In CA19-9-high PDAC (TCGA Firehose Legacy) and esophageal adenocarcinoma (TCGA PanCancer Atlas), LAT1 and FUT3 demonstrated an inverse correlation (pancreatic: Spearman = −0.21, *p* = 9.75 × 10^− 3^; esophageal: Spearman = −0.41, *p* = 9.13 × 10^− 9^; Fig. 2C and **D**)

TCGA LAT1–FUT3 analysis: CA19-9–producing tumors show lower LAT1 and higher FUT3; an inverse LAT1–FUT3 correlation is specific to PDAC and esophageal adenocarcinoma, implying limited BPA suitability in CA19-9-producing tumors.

### Synthesis of (fucose triacetate)-^**10**^BSH conjugate 2 and uptake of fucose-BSH into CA19-9-high tumors

**Fig. 3:**
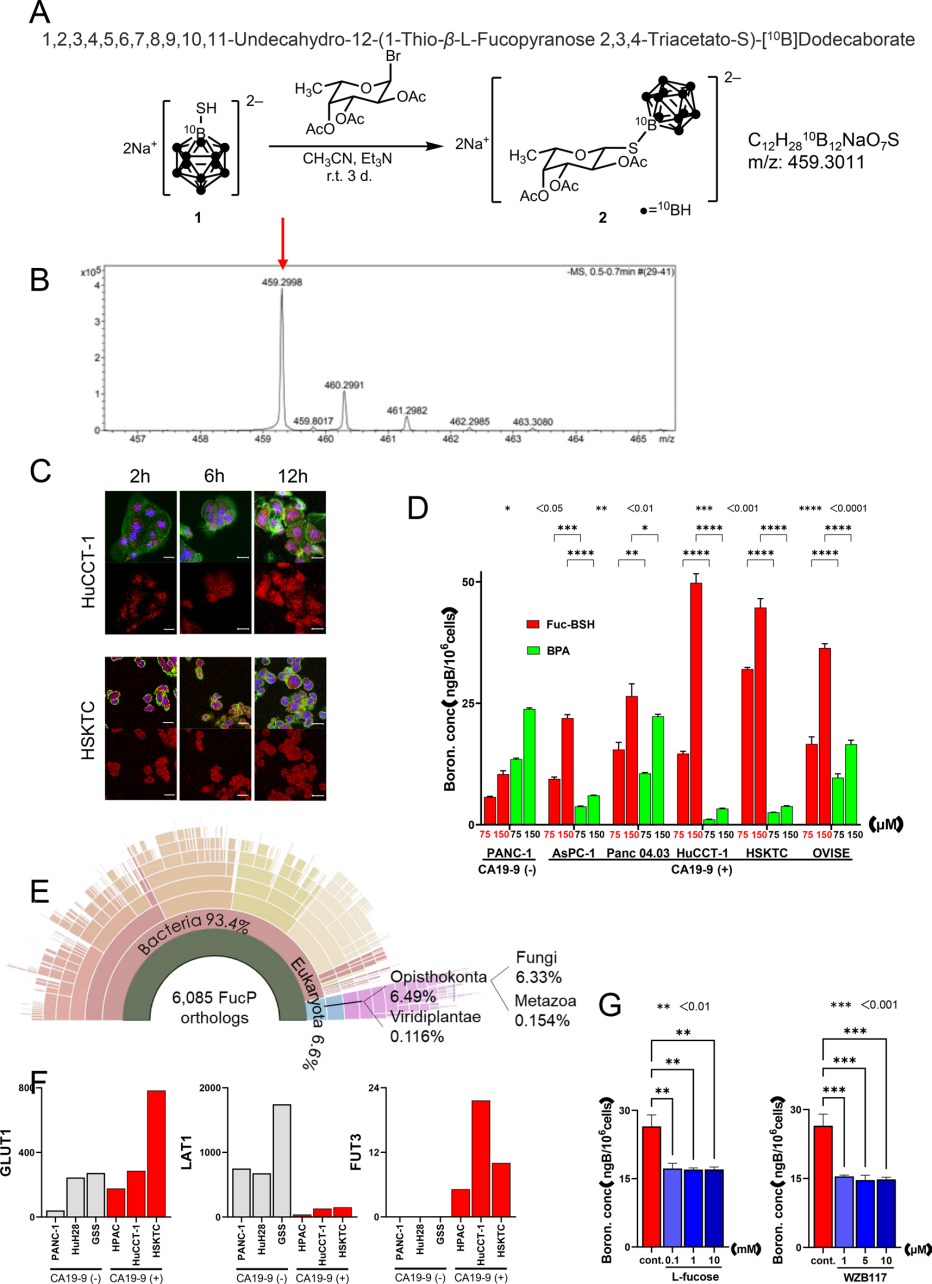
Synthesis and intracellular uptake of a novel fucose-conjugated boron compound (fucose-BSH). (**A**) synthetic scheme of (fucose triacetate)-^1^ 10BSH conjugate (compound 2). (**B**) ESI-TOF mass spectrometry confirming the molecular identity of compound 2. (**C**) confocal microscopy showing intracellular localization of fucose-BSH in CA19-9–positive cells at 2, 6, and 12 h (red: bsh; green: phalloidin; blue: nuclei; scale bar = 20 µm). (**D**) intracellular boron concentration measured by ICP-MS in CA19-9–positive and –negative cells after 24 h incubation with fucose-BSH or bpa (*n* = 4). (**E**) orthologous protein analysis of the bacterial L-fucose transporter (FucP) using EggNOG. (**F**) expression levels of GLUT1, LAT1, and FUT3 mRNA in CA19-9–positive and –negative cells from the ccle dataset. (**G**) boron accumulation in panc 04.03 cells (ng/10^6^ cells ± sem) after treatment with L-fucose (left) or GLUT1 inhibitor WZB117 (right), measured by ICP-MS (*n* = 4)

The treatment of 2,3,4-tri-O-acetyl-6-deoxy-α-L-galactopyranosyl bromide with ^10^B-labeled mercaptoundecahydrododecaborate **1** (^10^BSH) resulted in (fucose triacetate)-^10^BSH conjugate **2** in 15% yield (Fig. 3A). The structure of **2** was confirmed by ^1^H NMR (Supplementary Figure 2A), ESI-TOF mass spectrometry (Fig. 3B), and IR spectrometry (Supplementary Figure S2B). The ^1^H NMR spectrum of **2** showed the expected resonance for the acetyl groups of the fucose unit (δ 1.99, 2.13, and 2.21) and boron cluster cage (δ 0.8–1.8). ESI-TOF MS of **2** showed a molecular ion peak at *m/z* = 459.2998 ([M + Na]^–^; C_12_H_28_^10^B_12_NaO_7_S requires *m/z* = 459.3011).

CA19-9-high HuCCT-1 and HSKTC cells were treated with 150 µM Fucose-BSH. Confocal laser microscopy revealed subcellular localization in the cytoplasm and nucleus at 2 h, which was also observed at 6 and 12 h (Fig. 3C). Intracellular boron levels measured by ICP were: HuCCT-1:49.8 ± 1.9, HSKTC: 44.7 ± 1.8, OVISE: 36.4 ± 0.9, Panc 04.03:26.5 ± 2.5, AsPC-1:21.9 ± 0.7 ngB/10^6^ cells after 24 hours of 150 µM Fucose-BSH (Fig. 3D). In the case of BPA, these values were significantly lower. CA19-9-normal PANC-1 showed 10.4 ± 0.7 with Fucose-BSH and 23.8 ± 0.2 ngB/10^6^ cells with BPA. Cytotoxicity evaluations using WST-8 assays showed no significant differences between conditions (Supplementary Figure S3A).

FucP in E. coli facilitated L-fucose uptake, but no homologs were identified in humans, mice, frogs, or fish (Fig. 3E). Ortholog analysis showed FucP distribution mainly in prokaryotes but rarely in eukaryotes. GLUT1 is implicated in fucose uptake (Fig. 3F). GLUT1, LAT1, and FUT3 expression levels were analyzed in CCLE: HPAC: GLUT1:177.1, LAT1:39.3, FUT3:5.2; HuCCT-1: GLUT1:286.4, LAT1:130.4, FUT3:21.6; HSKTC: GLUT1:783.6, LAT1:151.3, and FUT3:10.0. CA19-9-normal cells showed significantly higher LAT1 expression, but lower FUT3 levels.

Fucose-BSH uptake in Panc 04.03 cells decreased after pretreatment with L-fucose or the GLUT1 inhibitor, WZB117. The boron concentration dropped from 26.5 ± 2.5 (control) to: 17.2 ± 1.2 (0.1 mM L-fucose), 17.0 ± 0.4 (1 mM L-fucose), 17.0 ± 0.5 (10 mM L-fucose), and to: 15.5 ± 0.3 (1 µM WZB117), 14.7 ± 1.0 (5 µM WZB117), 14.8 ± 0.5 ngB/10^6^ cells (10 µM WZB117) (Fig. 3G, Supplementary Figure S3B). These results suggest that fucose-BSH uptake is partially dependent on GLUT1 but not FucP.

Chemistry & uptake: We synthesized a first-in-class fucose-conjugated boron compound, verified stability, and showed efficient uptake in CA19-9–producing cells (high FUT3, low LAT1); competition with L-fucose and GLUT inhibition implicate GLUT-mediated transport.

#### *In vitro***results of neutron irradiation with fucose-BSH in CA19-9-normal or CA19-9-high cancer cells**

Before BNCT experiments, colony formation assays confirmed the radiotherapy resistance of six cell lines (SF2 after 2 Gy X-ray irradiation): PANC-1 (0.81), PK-45 H (0.75), Panc 04.03 (0.92), HSKTC (1.00), HuCCT-1 (0.92), and OVISE (0.66) (Supplementary Figure S4). These results demonstrate radiation resistance across all lines.

**Fig. 4:**
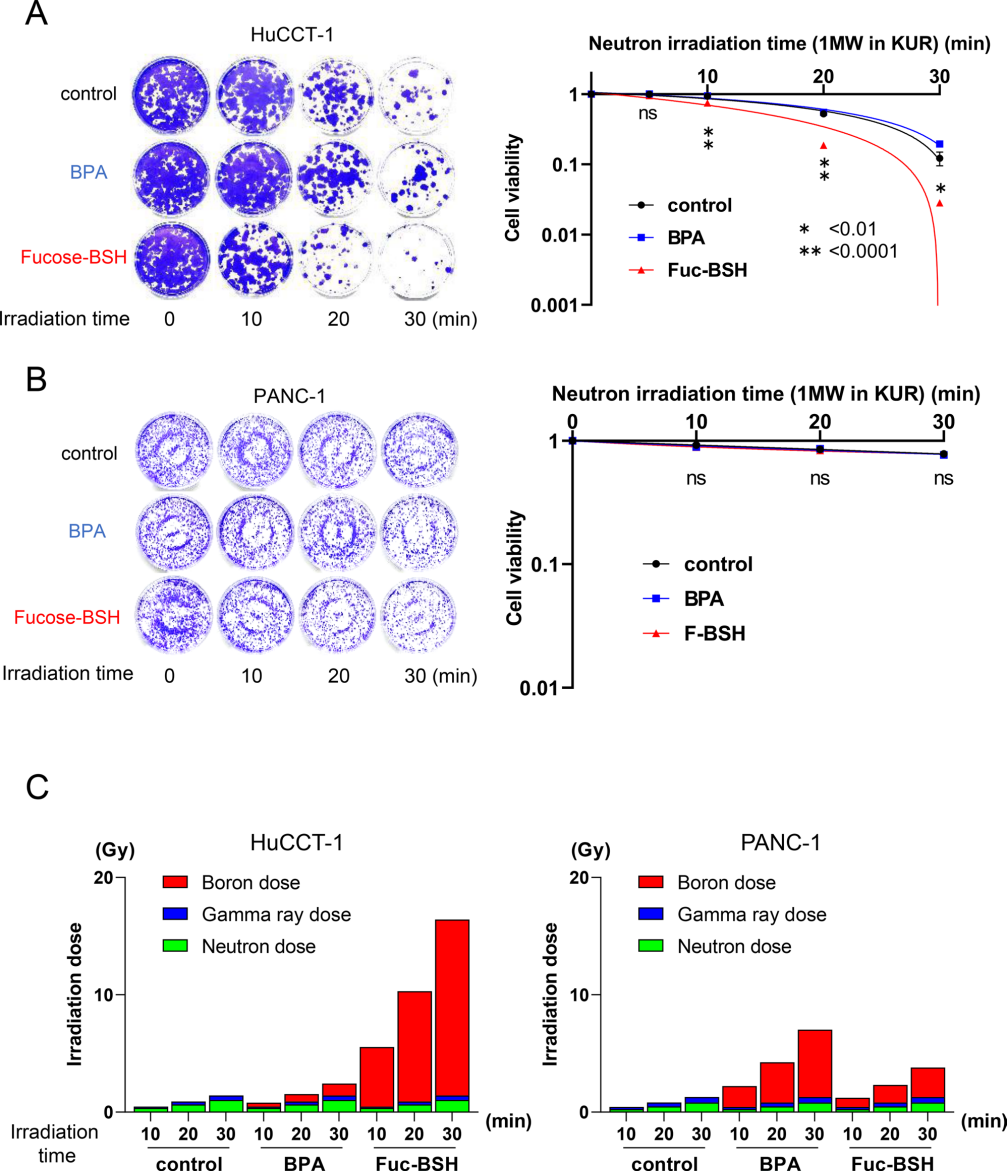
In vitro therapeutic efficacy of fucose-BSH–mediated BNCT in CA19-9–positive and –negative cell lines. (**A**) clonogenic survival of CA19-9–positive HuCCT-1 cells following fucose-BSH administration and neutron irradiation. (**B**) clonogenic survival of CA19-9–negative PANC-1 cells under the same conditions. (**C**) absorbed BNCT dose (gy) for each boron agent. Data are presented as mean ± sem (*n* = 4 per group). *p* < 0.01, *p* < 0.001. Neutron irradiation time (minutes) is shown on the X-axis

CA19-9-high HuCCT-1 and CA19-9-normal PANC-1 cells were treated with fucose-BSH or BPA, and irradiated with neutrons. Colony formation assays revealed fucose-BSH-BNCT significantly inhibited HuCCT-1 cell growth at neutron fluences of 1.39 × 10^1 2^, 2.59 × 10^1 2^, and 4.14 × 10^1 2^ n/cm^2^ (*p* < 0.01) (Fig. 4A; Supplementary Figure S5). In contrast, PANC-1 cells showed no significant effects at 3.29 × 10^1 2^ n/cm^2^ (Control: *p* = 0.95, BPA: *p* = 0.88) (Fig. 4B).

The estimated doses for fucose-BSH-BNCT in HuCCT-1 were 5.6 (10 min), 10.3 (20 min), and 16.5 Gy (30 min), while those in PANC-1 were 1.2 (10 min), 2.3 (20 min), and 3.8 Gy (30 min) (Fig. 4C; Supplementary Figure S5). These results underscore the strong antiproliferative effects of fucose-BSH-BNCT on CA19-9-high cells while sparing CA19-9-normal cells.

In vitro BNCT: Fucose-BSH–BNCT achieved greater killing in HuCCT-1 than in PANC-1, with higher estimated dose delivery despite similar X-ray resistance.

### Pharmacokinetics and BNCT results of fucose-BSH in the CA19-9-high HuCCT-1 cell mouse model

**Fig. 5:**
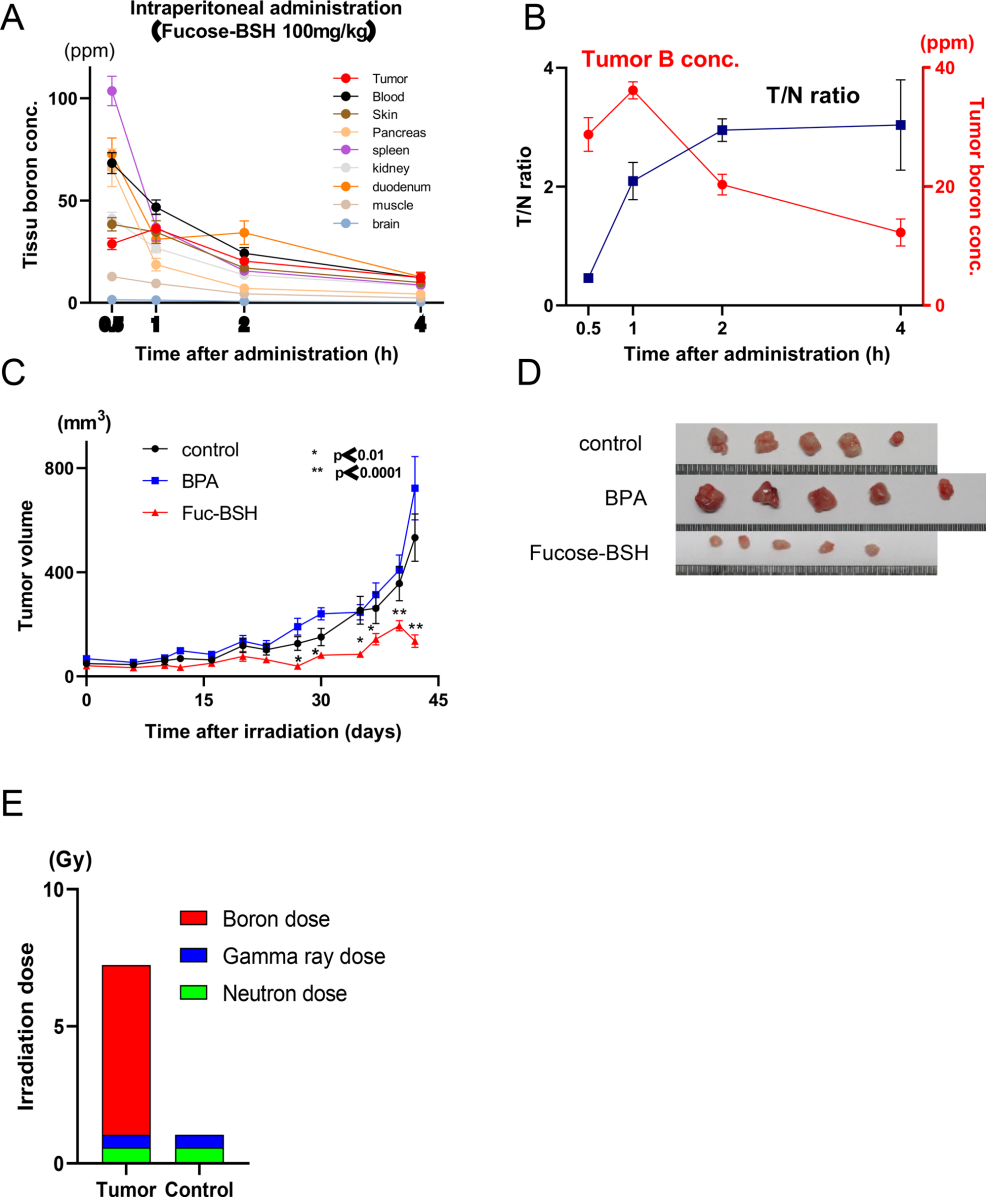
In vivo antitumor efficacy of fucose-BSH–mediated BNCT in the HuCCT-1 xenograft model. (**A**) boron concentration (ppm ± sem) in tumors and (**B**) tumor-to-normal tissue (pancreas) ratio in HuCCT-1–bearing mice (*n* = 4 per group). (**C**) tumor volume (mm^3^) following neutron irradiation (*p* < 0.01, *p* < 0.001; *n* = 5 per group). (**D**) Representative images of subcutaneous tumors at 42 days post-irradiation. (**E**) absorbed tumor dose (gy) in fucose-BSH-BNCT versus control groups

The antitumor effects of fucose-BSH were evaluated in vivo using HuCCT-1 cell transplanted BALB/c-nu/nu mice. Following intraperitoneal administration of fucose-BSH (100 mg/kg), tumors and tissues (blood, brain, kidney, duodenum, muscle, skin, pancreas, and spleen) were analyzed using ICP-MS over 0.5 to 4 hours. The highest boron concentration in tumors (36.2 ppm) was observed at 1 h, with skin and pancreas concentrations of 34.6 and 18.7 ppm, respectively, and a tumor/normal tissue ratio (T/N (pancreas)) of 2.1 (Fig. 5Aand **B**).

Neutron irradiation was performed 1 h after drug administration on day 14 post transplantation. The thermal neutron fluence ranged from 2.3–2.5 × 10^1 2^, and epi-thermal neutron fluence was 4.3–4.6 × 10^1 2^ n/cm^2^ (Supplementary Figure S6). Tumor volumes post-BNCT were monitored over time. On day 42 post-BNCT, tumor volumes were significantly smaller in the fucose-BSH-BNCT group (135.6 ± 24.1 mm^3^) than in the control (532.5 ± 91.2) and BPA-BNCT groups (722.6 ± 121.3) (Fig. 5C and **D**). The average irradiation doses were 7.24 for Fucose-BSH-BNCT and 1.05 Gy for the control (Fig. 5E).

These results highlight the potent and selective tumor-reducing effects of fucose-BSH-BNCT in CA19-9-high HuCCT-1 models, offering a safe and effective therapeutic strategy.

PK & in vivo BNCT: Fucose-BSH–BNCT significantly suppressed HuCCT-1 xenografts, attained favorable tumor-to-normal boron distribution, and surpassed BPA-BNCT, supporting safety and efficacy.

## Discussion

We previously reported the efficacy of precision BNCT for high-grade pancreatic cancer with glucose-BSH. In this study, we further demonstrated the efficacy of a new precision BNCT approach that incorporates tumor marker information. We successfully synthesized fucose-BSH by conjugating boron-containing drug BSH to the main component of the tumor marker CA19-9, fucose, and confirmed selective high uptake in high CA19-9 cancer groups, as well as high therapeutic effects in in vitro and in vivo neutron irradiation experiments. Additionally, we demonstrated that fucosyltransferase 3 (FUT3), which is required for CA19-9 synthesis, was inversely correlated with LAT1, the uptake target of BPA.

BPA-BNCT has shown efficacy in LAT1-high melanoma, head and neck cancer, and malignant brain tumors and is expected to be expanded further [9]. CA19-9 is a sugar compound in which fucose is bound to its precursor DUPAN2 by FUT3. We also identified an association between FUT3 expression and poor prognosis. Furthermore, we found that the expression patterns of FUT3 and LAT1, which are targets of BPA, were inversely correlated in the CA19-9-high malignancy group (Figs. 6C and D), indicating that fucose boron drugs are different targets of BPA. Importantly, this drug also showed efficacy in a group of CA19-9-high malignancies. In precision BNCT, we developed boron drugs with high tumor accumulation by targeting tumor markers such as CA19-9, in addition to highly expressed genes in target malignant tumors. This is the first attempt at drug discovery focusing on fucose, a component of CA19-9.

In certain malignancies, high CA19-9 levels are considered a poor prognostic factor, making the control of CA19-9 levels important for treatment [30–33]. To therapeutically reduce CA19-9 levels, a group of CA19-9-high malignancies (pancreatic cancer, colorectal cancer, bile duct cancer, and cancer of unknown primary) has been the focus of research using a CA19-9 neutralizing antibody (MVT-5873) [34]. Thirty-six patients with stage III or IV pancreatic cancer, one patient with colorectal cancer, and two patients with an unknown primary malignancy, all with high CA19-9 levels, participated in a phase I trial (NCT02672917). Among those with pancreatic cancer, three of six patients in the antibody treatment group with chemotherapy showed sustained tumor suppression at subnormal CA19-9 levels [25]. MVT-2163 (the radiolabeled high-affinity CA19-9 antibody HuMab-5B1) was successfully used in the world’s first clinical study for Immuno-PET Imaging in 12 pancreatic cancer patients with high CA19-9 levels [26]. Furthermore, ^177^Lu Human Monoclonal Antibody 5B1 (MVT-1075), a combination of ^177^Lu, a therapeutic nuclide in nuclear medicine, and a CA19-9 antibody, was developed and successfully used in a phase I study [27]. In addition, radioimmunotherapy using a CA19-9 antibody and ^225^Ac-labeled tetrazine radioligand has been reported [21]. However, the development of therapies that directly target CA19-9-producing malignancies is rare. Because there are currently no therapies that can directly target and treat malignancies with high CA19-9 levels, such as the fucose-BSH-BNCT method described here, our study is groundbreaking. Combination therapy with the aforementioned CA19-9 antibody is also possible.

In BNCT, the alpha particles and Li nuclei produced in the reaction between boron (^10^B) and neutrons are limited to 10 µm or less. Therefore, it is necessary to efficiently introduce boron drugs specifically into tumor cells. It is also necessary to simultaneously reduce its uptake into the surrounding normal cells. The minimum T/N of 2.0 has been used in clinical BNCT reports (e.g., recurrent meningioma) [35]. The minimum tumor ^10^ B for BNCT lethality is ~1 × 10^9^ atoms/cell (~20 µg/g). Our 1-h window met T/*N* ≥2.0 and tumor ^10^ B ≥ 20 ppm, aligning with these practical constraints [36]. To achieve this goal, various boron drug discovery approaches have been reported [37]. For boron drug discovery using polymeric DDS targeting cell surface markers, there are reports of immunoliposomes containing an anti-EGFR antibody bound to the liposome surface and boron drug BSH for malignant brain tumors with high EGFR expression levels [38]. Another study reported using a BPA-poly(vinyl alcohol) (PVA) complex for cancer cells by forming a BPA and PVA complex, which can prevent BPA from leaking out of the cytoplasm and thereby enhance its antitumor activity [39]. One study described the use of fucose-bound nanoparticles to encapsulate the anticancer drug cisplatin and treat pancreatic cancer [40]. However, there have been no reports other than ours on boron drug discovery using tumor markers and bioinformatics.

Although this study focused on CA19-9, the development of therapies targeting tumor markers is expanding, especially antibody therapies. For example, CA125-targeted maintenance therapy using anti-CA125 antibodies in ovarian cancer has been reported in clinical studies [41]. An anti-MUC16/CA125 antibody-bound nanotherapeutic implant drug delivery system improved the survival rate in an ovarian cancer mouse model [42]. In addition to carbohydrate antigens, photoimmunotherapy targeting cancer embryonic antigen (CEA) has been reported [43]. Targeted alpha-therapy (TAT) using anti-CEA antibodies and ^225^Ac, as well as increased intracellular delivery of the active metabolite of irinotecan (SN-38) bound to anti-CEA antibodies in an animal model of colon cancer, has been reported to be effective [44, 45]. Based on these findings, we propose that fucose-BSH-BNCT targets cell populations with high CA19-9 levels. This cell population may exhibit high FUT-3 expression levels. We believe that it is possible to further expand the potential of BNCT by focusing on other tumor markers and their associated gene expression. A boron drug discovery project focusing on CA19-9 synthesis is the first of its kind, emphasizing the importance of developing precise BNCT tailored to specific patient cancer genes and biomarkers.

Although the potential of fucose-BSH-BNCT was demonstrated in this study, it should be further explored in future studies. Analysis of boron distribution and radiation dose simulation is necessary to reasonably predict the radiobiological effects of BNCT [46]. Additionally, research on the development of molecular imaging methods such as ^18^F-FBPA, which evaluates BPA pharmacokinetics, must be pursued simultaneously [47]. We are currently investigating the pharmacokinetic evaluation of fucose-BSH by PET imaging with metal chelators (DOTA) to address this problem [48]. One of the most important advantages of BNCT is its minimal invasiveness; however, its minor effects on normal organs cannot be neglected. We consider this an important issue when considering the clinical application of BSH-BNCT. Targeting CA19-9-high malignant tumors requires irradiation of deep organs from the body surface, and neutron irradiation methods using open abdominal approaches should be considered in the future [29]. In conclusion, precision BNCT based on fucose-BSH represents a new treatment strategy for CA19-9-high cancers. CA19-9 is generated when FUT3 acts on the precursor DUPAN-2, resulting in fucose binding. Bioinformatics analysis of CA19-9-high cancers revealed elevated FUT3 expression. We developed a fucose-bound boron compound and demonstrated the antitumor efficacy of BNCT using this novel drug in CA19-9-high cancer cells. While fucose-based drugs and fucose targeting are important for targeting CA19-9–producing cancers, further elucidation of the uptake mechanism is also necessary. Combining fucose-BSH-BNCT with existing cancer therapies may provide a multidisciplinary treatment option for patients with CA19-9-high tumors and poor prognosis (Graphic abstract). We propose to validate Fucose-BSH–BNCT in patient-derived xenograft (PDX) models established from CA19-9–high tumors. Building on evidence that CA19-9 can activate EGFR signaling and promote malignant phenotypes [22], and given the scarcity of approaches that directly modulate CA19-9, we will test Fucose-BSH–BNCT as a selective locoregional modality within multimodal care. In pancreatic cancer, where markedly elevated CA19-9 often precludes resection, we will evaluate whether optimized-timing Fucose-BSH–BNCT can reduce tumor burden and circulating CA19-9 to expand eligibility for conversion to curative surgery.

Our precision BNCT is a drug design method based on multifaceted considerations in boron drug discovery, such as clear targets, targeted highly expressed tumor targets, and screening methods when using the drug. We are confident that this theory can be used not only in BNCT but also in other fields.

## Electronic supplementary material

Below is the link to the electronic supplementary material.

Supplementary material 1

Supplementary material 2

Supplementary material 3

Supplementary material 4

Supplementary material 5

Supplementary material 6

## Data Availability

Data will be made available upon request.

## References

[1] Locher GL. Biological effects and therapeutic possibilities of neutrons. Am J Roentgenol Radium Ther. 1936;36:1–13.

[2] Nedunchezhian K, Aswath N, Thiruppathy M, Thirugnanamurthy S. Boron neutron capture therapy - a literature review. J Clin Diagn Res. 2016;10:ZE01–04. 10.7860/JCDR/2016/19890.9024 PMC5296588 28209015

[3] Wongthai P, Hagiwara K, Miyoshi Y, Wiriyasermkul P, Wei L, Ohgaki R, Kato I, Hamase K, Nagamori S, Kanai Y. Boronophenylalanine, a boron delivery agent for boron neutron capture therapy, is transported by atb, LAT1 and LAT2. Cancer Sci. 2015;106:279–86. 10.1111/cas.12602 PMC4376436 25580517

[4] Hirose K, Konno A, Hiratsuka J, Yoshimoto S, Kato T, Ono K, Otsuki N, Hatazawa J, Tanaka H, Takayama K, et al. Boron neutron capture therapy using cyclotron-based epithermal neutron source and borofalan ((10)B) for recurrent or locally advanced head and neck cancer (JHN002): an open-label phase ii trial. Radiother Oncol. 2021;155:182–87. 10.1016/j.radonc.2020.11.001 33186684

[5] Miyatake S, Kawabata S, Kajimoto Y, Aoki A, Yokoyama K, Yamada M, Kuroiwa T, Tsuji M, Imahori Y, Kirihata M, et al. Modified boron neutron capture therapy for malignant gliomas performed using epithermal neutron and two boron compounds with different accumulation mechanisms: an efficacy study based on findings on neuroimages. J Neurosurg. 2005;103:1000–09. 10.3171/jns.2005.103.6.1000 16381186

[6] Mishima Y, Honda C, Ichihashi M, Obara H, Hiratsuka J, Fukuda H, Karashima H, Kobayashi T, Kanda K, Yoshino K. Treatment of malignant melanoma by single thermal neutron capture therapy with melanoma-seeking 10B-compound. Lancet. 1989;2:388–89. 10.1016/s0140-6736(89)90567-9 2569577

[7] Snyder HR, Reedy AJ, Lennarz WJ. Synthesis of aromatic boronic acids. Aldehydo boronic acids and a boronic acid analog of Tyrosine1. J Am Chem Soc. 1958;80:835–38.

[8] Kanai Y. Amino acid transporter LAT1 (SLC7A5) as a molecular target for cancer diagnosis and therapeutics. Pharmacol Ther. 2022;230. 10.1016/j.pharmthera.2021.107964 34390745

[9] Nakamura H. Historical development and current status of boron delivery agents for boron neutron capture therapy. Radioisotopes. 2015;64:47–58.

[10] Bhatt AN, Mathur R, Farooque A, Verma A, Dwarakanath BS. Cancer biomarkers - current perspectives. Indian J Med Res. 2010;132:129–49. 20716813

[11] Virji MA, Mercer DW, Herberman RB. Tumor markers in cancer diagnosis and prognosis. CA Cancer J Clin. 1988;38:104–26. 10.3322/canjclin.38.2.104 3127000

[12] Nagpal M, Singh S, Singh P, Chauhan P, Zaidi MA. Tumor markers: a diagnostic tool. Natl J Maxillofac Surg. 2016;7:17–20. 10.4103/0975-5950.196135 PMC5242068 28163473

[13] Sharma S. Tumor markers in clinical practice: general principles and guidelines. Indian J Med Paediatr Oncol. 2009;30:1–8. 10.4103/0971-5851.56328 PMC2902207 20668599

[14] Zhou Y, Tao L, Qiu J, Xu J, Yang X, Zhang Y, Tian X, Guan X, Cen X, Zhao Y. Tumor biomarkers for diagnosis, prognosis and targeted therapy. Signal Transduct Target Ther. 2024;9:132. 10.1038/s41392-024-01823-2 PMC11102923 38763973

[15] Lee T, Teng TZJ, Shelat VG. Carbohydrate antigen 19–9 - tumor marker: past, present, and future. World J Gastrointest Surg. 2020;12. 10.4240/wjgs.v12.i12.468 PMC7769746 33437400

[16] Koprowski H, Steplewski Z, Mitchell K, Herlyn M, Herlyn D, Fuhrer P. Colorectal carcinoma antigens detected by hybridoma antibodies. Somatic Cell Genet. 1979;5:957–71. 10.1007/BF01542654 94699

[17] Araki E, Kamiya K, Kawauchi A, Koike T, Takatsuka J, Takeuchi S, Namba M, Shida S, Ishiguro T, Tomoyoshi T, et al. Clinical assessment of urinary free L-fucose levels. Rinsho Byori. 1992;40:868–74. 1404961

[18] Hartwig W, Strobel O, Hinz U, Fritz S, Hackert T, Roth C, Buchler MW, Werner J. CA19-9 in potentially resectable pancreatic cancer: perspective to adjust surgical and perioperative therapy. Ann Surg Oncol. 2013;20:2188–96. 10.1245/s10434-012-2809-1 23247983

[19] Boone BA, Steve J, Krasinskas AM, Zureikat AH, Lembersky BC, Gibson MK, Stoller RG, Zeh HJ, Bahary N. Outcomes with FOLFIRINOX for borderline resectable and locally unresectable pancreatic cancer. J Surg Oncol. 2013;108:236–41. 23955427 10.1002/jso.23392 PMC3816713

[20] Hata S, Sakamoto Y, Yamamoto Y, Nara S, Esaki M, Shimada K, Kosuge T. Prognostic impact of postoperative serum ca 19–9 levels in patients with resectable pancreatic cancer. Ann Surg Oncol. 2012;19:636–41. 21863360 10.1245/s10434-011-2020-9

[21] Poty S, Carter LM, Mandleywala K, Membreno R, Abdel-Atti D, Ragupathi A, Scholz WW, Zeglis BM, Lewis JS. Leveraging bioorthogonal click chemistry to improve Ac-radioimmunotherapy of pancreatic ductal adenocarcinoma. Clin Cancer Res. 2019;25(3):868–880. 10.1158/1078-0432.CCR-18-1650 PMC6343144 30352909

[22] Engle DD, Tiriac H, Rivera KD, Pommier A, Whalen S, Oni TE, Alagesan B, Lee EJ, Yao MA, Lucito MS, et al. The glycan CA19-9 promotes pancreatitis and pancreatic cancer in mice. Science. 2019;364:1156–62. 31221853 10.1126/science.aaw3145 PMC6705393

[23] Wu Y, Liu F, Luo S, Yin X, He D, Liu J, Yue Z, Song J. Co-expression of key gene modules and pathways of human breast cancer cell lines. Biosci Rep. 2019;39. 10.1042/BSR20181925 PMC6639467 31285391

[24] Gebauer F, Wicklein D, Stübke K, Nehmann N, Schmidt A, Salamon J, Peldschus K, Nentwich MF, Adam G, Tolstonog G, et al. Selectin binding is essential for peritoneal carcinomatosis in a xenograft model of human pancreatic adenocarcinoma in pfp/rag2 mice. Gut. 2013;62:741–50. 22490524 10.1136/gutjnl-2011-300629

[25] O’Reilly EM, Wang J-Z, Yu KH, Lowery MA, Varghese AM, Bendell JC, Borazanci EH, Estrella H, Fowler K, Hoskins M, et al. Abstract LB-B25: preliminary phase I data comparing HuMab-5B1 (MVT-5873), a monoclonal antibody targeting sLea, as a single agent and in combination with first line nab-paclitaxel and gemcitabine in patients with CA19-9 positive pancreatic cancer. Mol Cancer Ther. 2018;17:LB-B25–LB-B25.

[26] Lohrmann C, O’Reilly EM, O’Donoghue JA, Pandit-Taskar N, Carrasquillo JA, Lyashchenko SK, Ruan S, Teng R, Scholz W, Maffuid PW, et al. Retooling a Blood-based Biomarker: phase I assessment of the high-affinity CA19-9 antibody HuMab-5B1 for immuno-PET imaging of pancreatic cancer. Clin Cancer Res. 2019;25:7014–23. 31540979 10.1158/1078-0432.CCR-18-3667 PMC7052809

[27] O’Reilly EA, Lohrmann C, O’Donoghue JA, Borazanci E, Estrella H, Teng R, Melink T, Dorr K, Kearns C, Peterson M, et al. Abstract CT140: phase I dose escalation study of 177Lu-HuMab-5B1 (MVT-1075) in combination with MVT-5873 as radioimmunotherapy (RIT) in subjects with relapsed/refractory pancreatic cancer or other CA19-9+ malignancies. Cancer Res. 2018;78:CT140–140.

[28] Schneider CA, Rasband WS, Eliceiri KW. Nih image to ImageJ: 25 years of image analysis. Nat Methods. 2012;9:671–75. 22930834 10.1038/nmeth.2089 PMC5554542

[29] Fujimoto T, Teraishi F, Kanehira N, Tajima T, Sakurai Y, Kondo N, Yamagami M, Kuwada A, Morihara A, Kitamatsu M, et al. BNCT pancreatic cancer treatment strategy with glucose-conjugated boron drug. Biomaterials. 2024;309:122605. 38754291 10.1016/j.biomaterials.2024.122605

[30] Hahn F, Muller L, Jungmann F, Mahringer-Kunz A, Tanyildizi Y, Duber C, Galle PR, Weinmann A, Kloeckner R. Survival prediction for patients with non-resectable intrahepatic cholangiocarcinoma undergoing chemotherapy: a retrospective analysis comparing the tumor marker ca 19–9 with cross-sectional imaging. J Cancer researchclin Oncol. 2020;146:1883–90. 10.1007/s00432-020-03200-2 PMC7256028 32232655

[31] Li Z, Zhuo Q, Li B, Liu M, Chen C, Shi Y, Xu W, Liu W, Ji S, Yu X, Xu X. Feasibility of laparoscopic versus open pancreatoduodenectomy following neoadjuvant chemotherapy for borderline resectable pancreatic cancer: a retrospective cohort study. World J Surg Oncol. 2024;22:1. 38169384 10.1186/s12957-023-03277-2 PMC10759588

[32] Wang F, Yuan X, Jia J, Bi X, Zhou Z, Zhou Q, Li X, Luo C, Deng M, Yi L, et al. Apatinib monotherapy for chemotherapy-refractory metastatic colorectal cancer: a multi-centre, single-arm, prospective study. Sci Rep. 2020;10:6058. 32269247 10.1038/s41598-020-62961-5 PMC7142071

[33] Zhu J, Jiang L, Wen H, Bi R, Wu X, Ju X. Prognostic value of serum CA19-9 and perioperative CA-125 levels in ovarian clear cell carcinoma. Int J Gynecol Cancer. 2018;28:1108–16. 29781825 10.1097/IGC.0000000000001293

[34] Gupta S, McDonald JD, Ayabe R, Khan TM, Gamble LA, Sinha S, Hannah C, Blakely AM, Davis JL, Hernandez JM. Targeting ca 19–9 with a humanized monoclonal antibody at the time of surgery may decrease recurrence rates for patients undergoing resections for pancreatic cancer, cholangiocarcinoma and metastatic colorectal cancer. J Gastrointest Oncol. 2020;11:231–35. 32399263 10.21037/jgo.2020.02.01 PMC7212097

[35] Lan TL, Lin CF, Lee YY, Chang FC, Lin SC, Chou FI, Peir JJ, Pan PS, Chen JK, Lai LH, et al. Recurrent meningioma treated with boron neutron capture therapy: a feasibility study with dosimetric and clinical correlates. J Neurooncol. 2025;175:879–86. 40745077 10.1007/s11060-025-05184-w PMC12420710

[36] Dymova MA, Taskaev SY, Richter VA, Kuligina EV. Boron neutron capture therapy: current status and future perspectives. Cancer Commun (lond). 2020;40:406–21. 32805063 10.1002/cac2.12089 PMC7494062

[37] Michiue H, Kitamatsu M, Fukunaga A, Tsuboi N, Fujimura A, Matsushita H, Igawa K, Kasai T, Kondo N, Matsui H, Furuya S. Self-assembling A6K peptide nanotubes as a mercaptoundecahydrododecaborate (BSH) delivery system for boron neutron capture therapy (BNCT). J Control Release. 2021;330:788–96. 33188824 10.1016/j.jconrel.2020.11.001

[38] Feng B, Tomizawa K, Michiue H, Miyatake S, Han XJ, Fujimura A, Seno M, Kirihata M, Matsui H. Delivery of sodium borocaptate to glioma cells using immunoliposome conjugated with anti-EGFR antibodies by ZZ-His. Biomaterials. 2009;30:1746–55. 19121537 10.1016/j.biomaterials.2008.12.010

[39] Nomoto T, Inoue Y, Yao Y, Suzuki M, Kanamori K, Takemoto H, Matsui M, Tomoda K, Nishiyama N. Poly(vinyl alcohol) boosting therapeutic potential of p-boronophenylalanine in neutron capture therapy by modulating metabolism. Sci Adv. 2020;6:eaaz 1722. 10.1126/sciadv.aaz1722 PMC6976296 32010792

[40] Yoshida M, Takimoto R, Murase K, Sato Y, Hirakawa M, Tamura F, Sato T, Iyama S, Osuga T, Miyanishi K, et al. Targeting anticancer drug delivery to pancreatic cancer cells using a fucose-bound nanoparticle approach. PLoS One. 2012;7:e39545. 10.1371/journal.pone.0039545 PMC3394772 22808043

[41] Mei L, Hou Q, Fang F, Wang H. The antibody-based CA125-targeted maintenance therapy for the epithelial ovarian cancer: a meta-analysis. Eur J Gynaecol Oncol. 2016;37:455–60. 29894066

[42] Pantshwa JM, Rhoda K, Clift SJ, Pradeep P, Choonara YE, Kumar P, du Toit LC, Penny C, Pillay V. Chemotherapeutic efficacy of implantable antineoplastic-treatment protocols in an optimal mouse Model for human ovarian carcinoma cell targeting. Int J Mol Sci. 2018;19. 10.3390/ijms19103030 PMC6213745 30287783

[43] Shirasu N, Yamada H, Shibaguchi H, Kuroki M, Kuroki M. Potent and specific antitumor effect of CEA-targeted photoimmunotherapy. Int. J. Cancer. 2014;135:2697–710. 10.1002/ijc.28907 24740257

[44] Minnix M, Kujawski M, Poku E, Yazaki PJ, Wong JY, Shively JE. Improved tumor responses with sequential targeted alpha-particles followed by interleukin 2 Immunocytokine therapies in treatment of CEA-Positive breast and colon tumors in cea transgenic mice. J Nucl Med. 2022;63:1859–64. 10.2967/jnumed.122.264126 PMC9730924 35772959

[45] Nessler I, Rubahamya B, Kopp A, Hofsess S, Cardillo TM, Sathyanarayan N, Donnell J, Govindan SV, Thurber GM. Improving intracellular delivery of an antibody-drug conjugate targeting carcinoembryonic antigen increases efficacy at clinically relevant doses in vivo. Mol Cancer Ther. 2024;23:343–53. 10.1158/1535-7163.MCT-23-0437 PMC10932886 37913500

[46] Ono K, Tanaka H, Tamari Y, Watanabe T, Suzuki M, Masunaga SI. Proposal for determining absolute biological effectiveness of boron neutron capture therapy-the effect of 10B(n, alpha)7Li dose can be predicted from the nucleocytoplasmic ratio or the cell size. J Radiat Res. 2019;60:29–36. 10.1093/jrr/rry080 PMC6373679 30395286

[47] Ishiwata K, Ido T, Kawamura M, Kubota K, Ichihashi M, Mishima Y. 4-Borono-2-[18F]fluoro-D, L-phenylalanine as a target compound for boron neutron capture therapy: tumor imaging potential with positron emission tomography. Int J RAD Appl Instrum B. 1991;18:745–51. 10.1016/0883-2897(91)90013-b 1787083

[48] Iguchi Y, Michiue H, Kitamatsu M, Hayashi Y, Takenaka F, Nishiki T, Matsui H. Tumor-specific delivery of BSH-3R for boron neutron capture therapy and positron emission tomography imaging in a mouse brain tumor model. Biomaterials. 2015;56:10–17. 10.1016/j.biomaterials.2015.03.061 25934274

