# Supplementary material for: Tumor marker–guided precision BNCT for CA19-9–positive cancers: a new paradigm in molecularly targeted chemoradiation therapy
Source: J Transl Med. 2025 Dec 8;23:1387. doi: 10.1186/s12967-025-07349-7 (PMC12683832; doi:10.1186/s12967-025-07349-7)
Supplement: Supplementary file 1 — Supplementary material 1 [file 12967_2025_7349_MOESM1_ESM.pptx]

## Slide 1
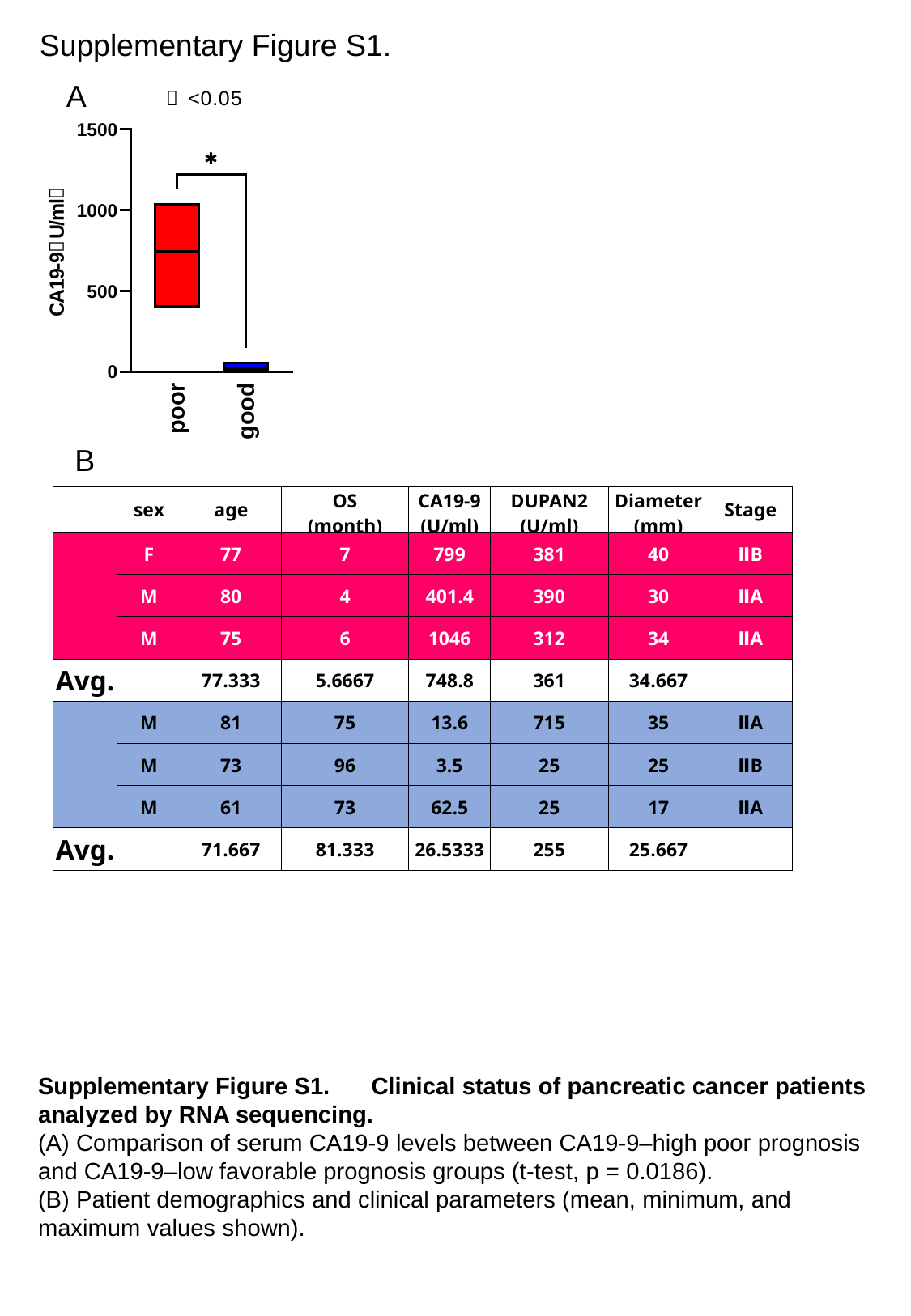

Supplementary Figure S1.
A
 B
| | sex | age | OS (month) | CA19-9 (U/ml) | DUPAN2 (U/ml) | Diameter (mm) | Stage |
| --- | --- | --- | --- | --- | --- | --- | --- |
| | F | 77 | 7 | 799 | 381 | 40 | ⅡB |
| | M | 80 | 4 | 401.4 | 390 | 30 | ⅡA |
| | M | 75 | 6 | 1046 | 312 | 34 | ⅡA |
| Avg. | | 77.333 | 5.6667 | 748.8 | 361 | 34.667 | |
| | M | 81 | 75 | 13.6 | 715 | 35 | ⅡA |
| | M | 73 | 96 | 3.5 | 25 | 25 | ⅡB |
| | M | 61 | 73 | 62.5 | 25 | 17 | ⅡA |
| Avg. | | 71.667 | 81.333 | 26.5333 | 255 | 25.667 | |
Supplementary Figure S1.　 Clinical status of pancreatic cancer patients analyzed by RNA sequencing.
(A) Comparison of serum CA19-9 levels between CA19-9–high poor prognosis and CA19-9–low favorable prognosis groups (t-test, p = 0.0186).
(B) Patient demographics and clinical parameters (mean, minimum, and maximum values shown).
