# Supplementary material for: Tumor marker–guided precision BNCT for CA19-9–positive cancers: a new paradigm in molecularly targeted chemoradiation therapy
Source: J Transl Med. 2025 Dec 8;23:1387. doi: 10.1186/s12967-025-07349-7 (PMC12683832; doi:10.1186/s12967-025-07349-7)
Supplement: Supplementary file 2 — Supplementary material 2 [file 12967_2025_7349_MOESM2_ESM.pptx]

## Slide 1
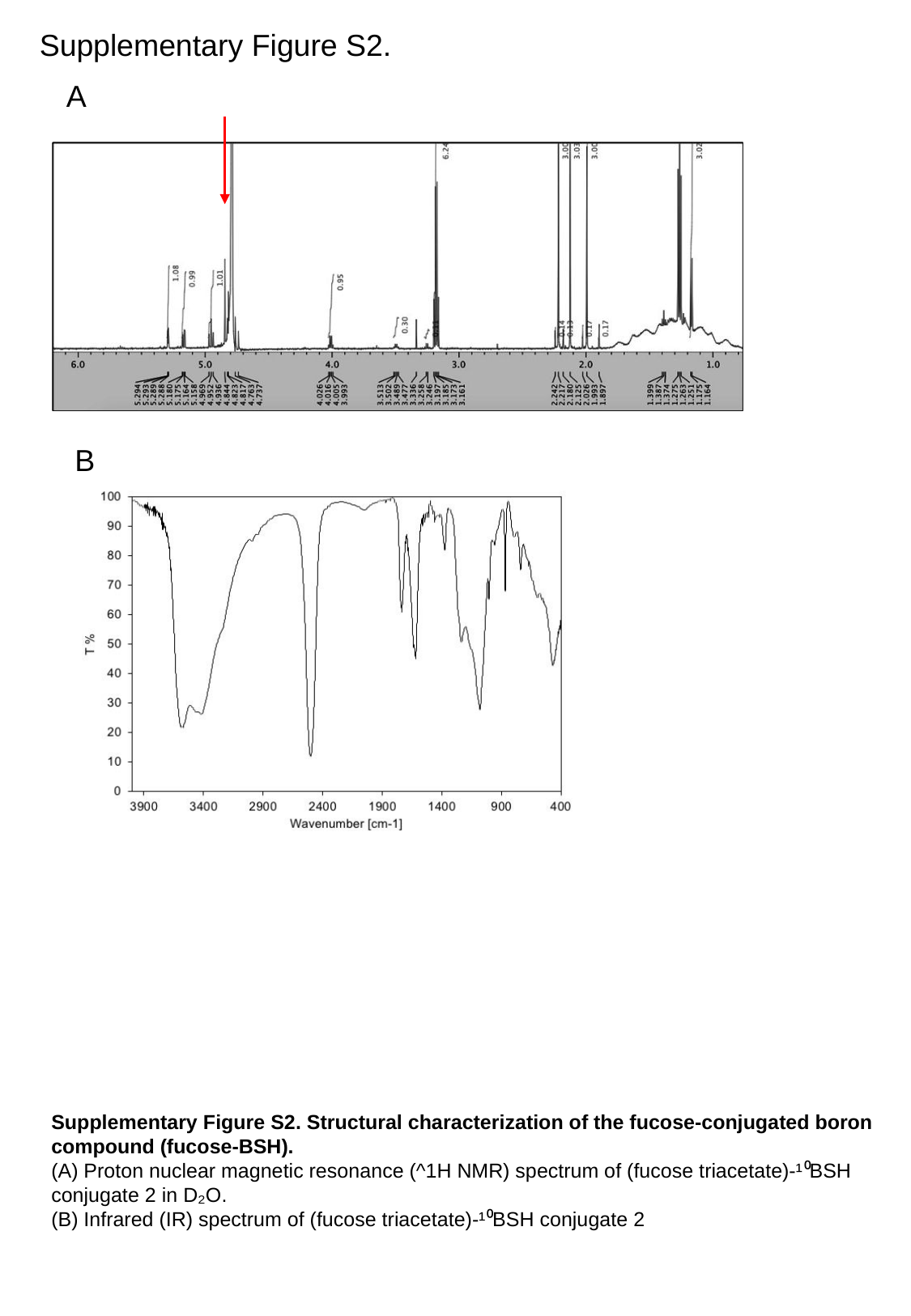

Supplementary Figure S2.
A
 B
Supplementary Figure S2. Structural characterization of the fucose-conjugated boron compound (fucose-BSH).
(A) Proton nuclear magnetic resonance (^1H NMR) spectrum of (fucose triacetate)-¹⁰BSH conjugate 2 in D₂O.
(B) Infrared (IR) spectrum of (fucose triacetate)-¹⁰BSH conjugate 2
