# Supplementary material for: Tumor marker–guided precision BNCT for CA19-9–positive cancers: a new paradigm in molecularly targeted chemoradiation therapy
Source: J Transl Med. 2025 Dec 8;23:1387. doi: 10.1186/s12967-025-07349-7 (PMC12683832; doi:10.1186/s12967-025-07349-7)
Supplement: Supplementary file 3 — Supplementary material 3 [file 12967_2025_7349_MOESM3_ESM.pptx]

## Slide 1
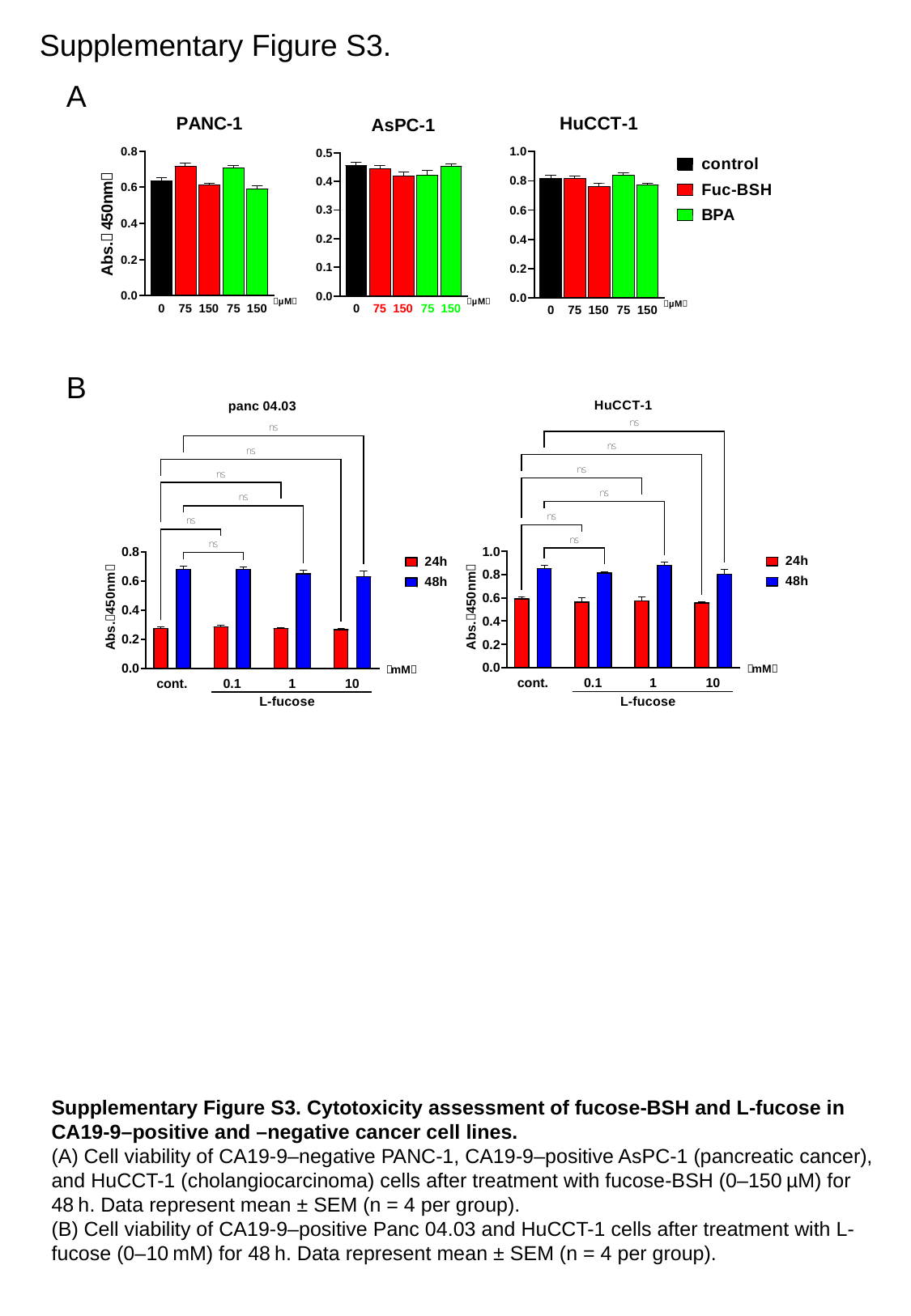

Supplementary Figure S3.
A
B
Supplementary Figure S3. Cytotoxicity assessment of fucose-BSH and L-fucose in CA19-9–positive and –negative cancer cell lines.
(A) Cell viability of CA19-9–negative PANC-1, CA19-9–positive AsPC-1 (pancreatic cancer), and HuCCT-1 (cholangiocarcinoma) cells after treatment with fucose-BSH (0–150 µM) for 48 h. Data represent mean ± SEM (n = 4 per group).
(B) Cell viability of CA19-9–positive Panc 04.03 and HuCCT-1 cells after treatment with L-fucose (0–10 mM) for 48 h. Data represent mean ± SEM (n = 4 per group).
