# Supplementary material for: Tumor marker–guided precision BNCT for CA19-9–positive cancers: a new paradigm in molecularly targeted chemoradiation therapy
Source: J Transl Med. 2025 Dec 8;23:1387. doi: 10.1186/s12967-025-07349-7 (PMC12683832; doi:10.1186/s12967-025-07349-7)
Supplement: Supplementary file 4 — Supplementary material 4 [file 12967_2025_7349_MOESM4_ESM.pptx]

## Slide 1
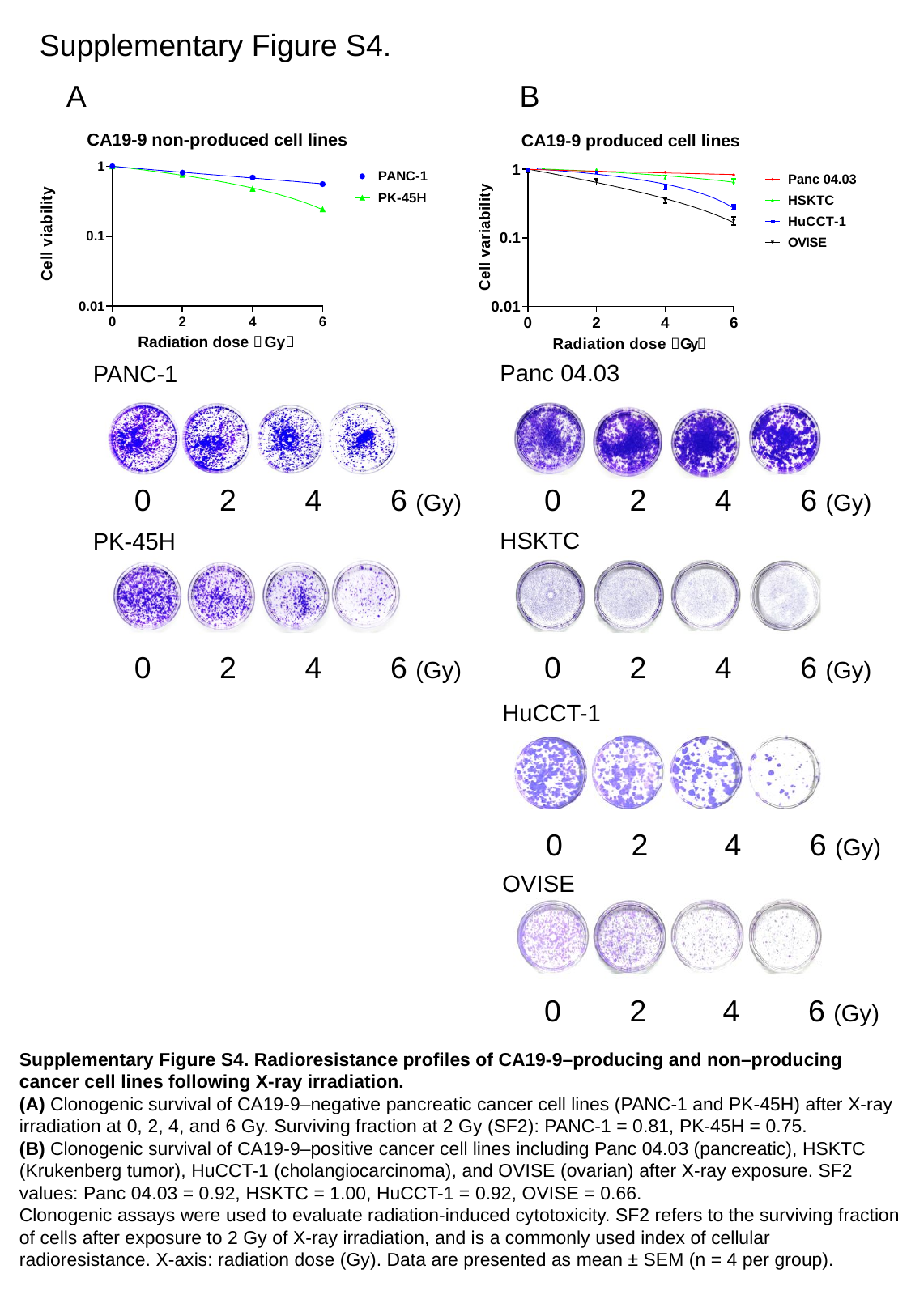

Supplementary Figure S4.
A　　　　　　　　　　　　　 B
Panc 04.03
PANC-1
 0　 2　 4　 6 (Gy)
 0　 2　 4　 6 (Gy)
HSKTC
PK-45H
 0　 2　 4　 6 (Gy)
 0　 2　 4　 6 (Gy)
HuCCT-1
 0　 2　 4　 6 (Gy)
OVISE
 0　 2　 4　 6 (Gy)
Supplementary Figure S4. Radioresistance profiles of CA19-9–producing and non–producing cancer cell lines following X-ray irradiation.
(A) Clonogenic survival of CA19-9–negative pancreatic cancer cell lines (PANC-1 and PK-45H) after X-ray irradiation at 0, 2, 4, and 6 Gy. Surviving fraction at 2 Gy (SF2): PANC-1 = 0.81, PK-45H = 0.75.
(B) Clonogenic survival of CA19-9–positive cancer cell lines including Panc 04.03 (pancreatic), HSKTC (Krukenberg tumor), HuCCT-1 (cholangiocarcinoma), and OVISE (ovarian) after X-ray exposure. SF2 values: Panc 04.03 = 0.92, HSKTC = 1.00, HuCCT-1 = 0.92, OVISE = 0.66.
Clonogenic assays were used to evaluate radiation-induced cytotoxicity. SF2 refers to the surviving fraction of cells after exposure to 2 Gy of X-ray irradiation, and is a commonly used index of cellular radioresistance. X-axis: radiation dose (Gy). Data are presented as mean ± SEM (n = 4 per group).
