# Supplementary material for: Tumor marker–guided precision BNCT for CA19-9–positive cancers: a new paradigm in molecularly targeted chemoradiation therapy
Source: J Transl Med. 2025 Dec 8;23:1387. doi: 10.1186/s12967-025-07349-7 (PMC12683832; doi:10.1186/s12967-025-07349-7)
Supplement: Supplementary file 5 — Supplementary material 5 [file 12967_2025_7349_MOESM5_ESM.pptx]

## Slide 1
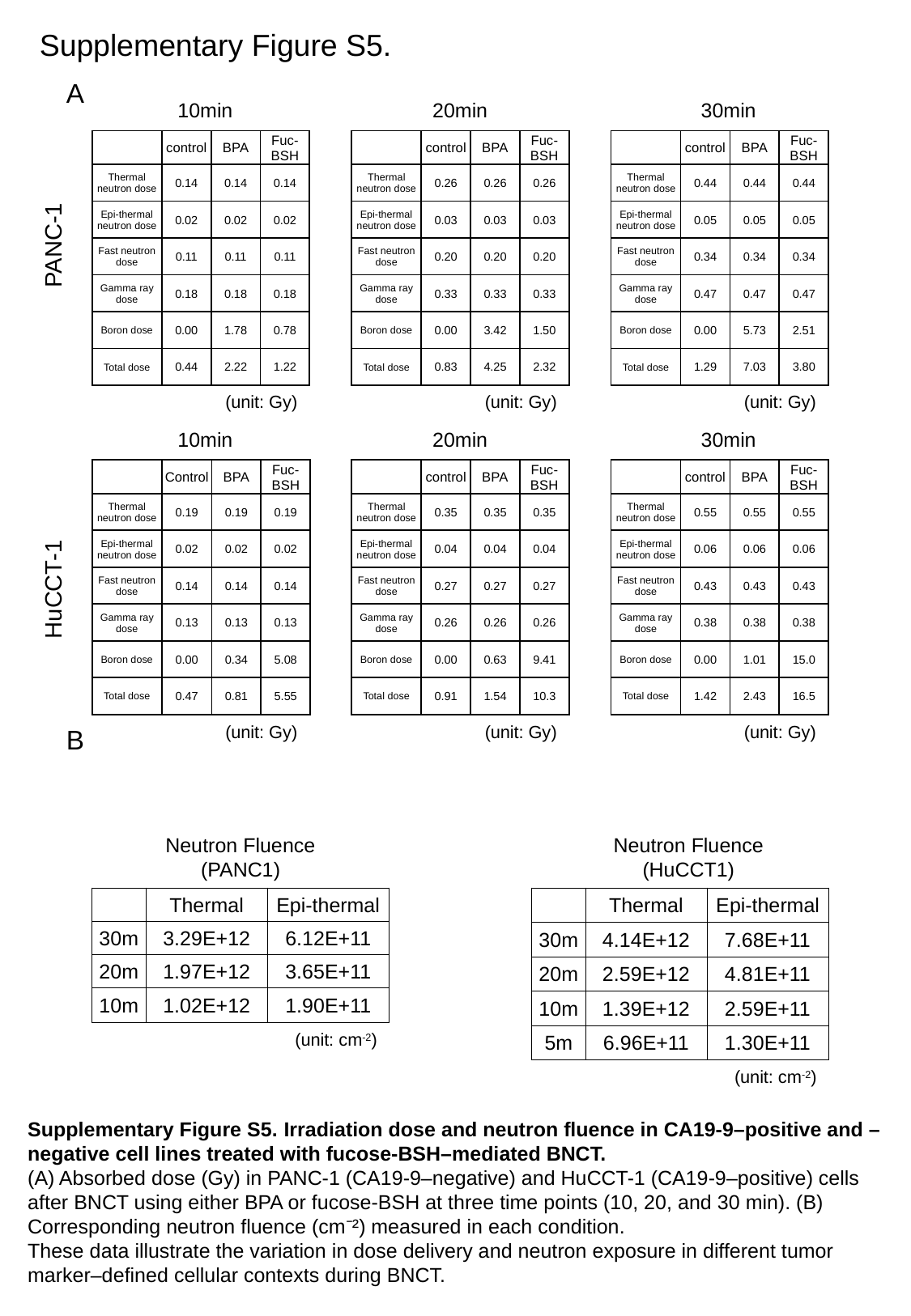

Supplementary Figure S5.
A
B
20min
30min
10min
| | control | BPA | Fuc-BSH |
| --- | --- | --- | --- |
| Thermal neutron dose | 0.14 | 0.14 | 0.14 |
| Epi-thermal neutron dose | 0.02 | 0.02 | 0.02 |
| Fast neutron dose | 0.11 | 0.11 | 0.11 |
| Gamma ray dose | 0.18 | 0.18 | 0.18 |
| Boron dose | 0.00 | 1.78 | 0.78 |
| Total dose | 0.44 | 2.22 | 1.22 |
| | control | BPA | Fuc-BSH |
| --- | --- | --- | --- |
| Thermal neutron dose | 0.26 | 0.26 | 0.26 |
| Epi-thermal neutron dose | 0.03 | 0.03 | 0.03 |
| Fast neutron dose | 0.20 | 0.20 | 0.20 |
| Gamma ray dose | 0.33 | 0.33 | 0.33 |
| Boron dose | 0.00 | 3.42 | 1.50 |
| Total dose | 0.83 | 4.25 | 2.32 |
| | control | BPA | Fuc-BSH |
| --- | --- | --- | --- |
| Thermal neutron dose | 0.44 | 0.44 | 0.44 |
| Epi-thermal neutron dose | 0.05 | 0.05 | 0.05 |
| Fast neutron dose | 0.34 | 0.34 | 0.34 |
| Gamma ray dose | 0.47 | 0.47 | 0.47 |
| Boron dose | 0.00 | 5.73 | 2.51 |
| Total dose | 1.29 | 7.03 | 3.80 |
PANC-1
(unit: Gy)
(unit: Gy)
(unit: Gy)
20min
30min
10min
| | Control | BPA | Fuc-BSH |
| --- | --- | --- | --- |
| Thermal neutron dose | 0.19 | 0.19 | 0.19 |
| Epi-thermal neutron dose | 0.02 | 0.02 | 0.02 |
| Fast neutron dose | 0.14 | 0.14 | 0.14 |
| Gamma ray dose | 0.13 | 0.13 | 0.13 |
| Boron dose | 0.00 | 0.34 | 5.08 |
| Total dose | 0.47 | 0.81 | 5.55 |
| | control | BPA | Fuc-BSH |
| --- | --- | --- | --- |
| Thermal neutron dose | 0.35 | 0.35 | 0.35 |
| Epi-thermal neutron dose | 0.04 | 0.04 | 0.04 |
| Fast neutron dose | 0.27 | 0.27 | 0.27 |
| Gamma ray dose | 0.26 | 0.26 | 0.26 |
| Boron dose | 0.00 | 0.63 | 9.41 |
| Total dose | 0.91 | 1.54 | 10.3 |
| | control | BPA | Fuc-BSH |
| --- | --- | --- | --- |
| Thermal neutron dose | 0.55 | 0.55 | 0.55 |
| Epi-thermal neutron dose | 0.06 | 0.06 | 0.06 |
| Fast neutron dose | 0.43 | 0.43 | 0.43 |
| Gamma ray dose | 0.38 | 0.38 | 0.38 |
| Boron dose | 0.00 | 1.01 | 15.0 |
| Total dose | 1.42 | 2.43 | 16.5 |
HuCCT-1
(unit: Gy)
(unit: Gy)
(unit: Gy)
Neutron Fluence
(HuCCT1)
Neutron Fluence
(PANC1)
| | Thermal | Epi-thermal |
| --- | --- | --- |
| 30m | 3.29E+12 | 6.12E+11 |
| 20m | 1.97E+12 | 3.65E+11 |
| 10m | 1.02E+12 | 1.90E+11 |
| | Thermal | Epi-thermal |
| --- | --- | --- |
| 30m | 4.14E+12 | 7.68E+11 |
| 20m | 2.59E+12 | 4.81E+11 |
| 10m | 1.39E+12 | 2.59E+11 |
| 5m | 6.96E+11 | 1.30E+11 |
(unit: cm-2)
(unit: cm-2)
Supplementary Figure S5. Irradiation dose and neutron fluence in CA19-9–positive and –negative cell lines treated with fucose-BSH–mediated BNCT.
(A) Absorbed dose (Gy) in PANC-1 (CA19-9–negative) and HuCCT-1 (CA19-9–positive) cells after BNCT using either BPA or fucose-BSH at three time points (10, 20, and 30 min). (B) Corresponding neutron fluence (cm⁻²) measured in each condition.
These data illustrate the variation in dose delivery and neutron exposure in different tumor marker–defined cellular contexts during BNCT.
