# Supplementary material for: Tumor marker–guided precision BNCT for CA19-9–positive cancers: a new paradigm in molecularly targeted chemoradiation therapy
Source: J Transl Med. 2025 Dec 8;23:1387. doi: 10.1186/s12967-025-07349-7 (PMC12683832; doi:10.1186/s12967-025-07349-7)
Supplement: Supplementary file 6 — Supplementary material 6 [file 12967_2025_7349_MOESM6_ESM.pptx]

## Slide 1
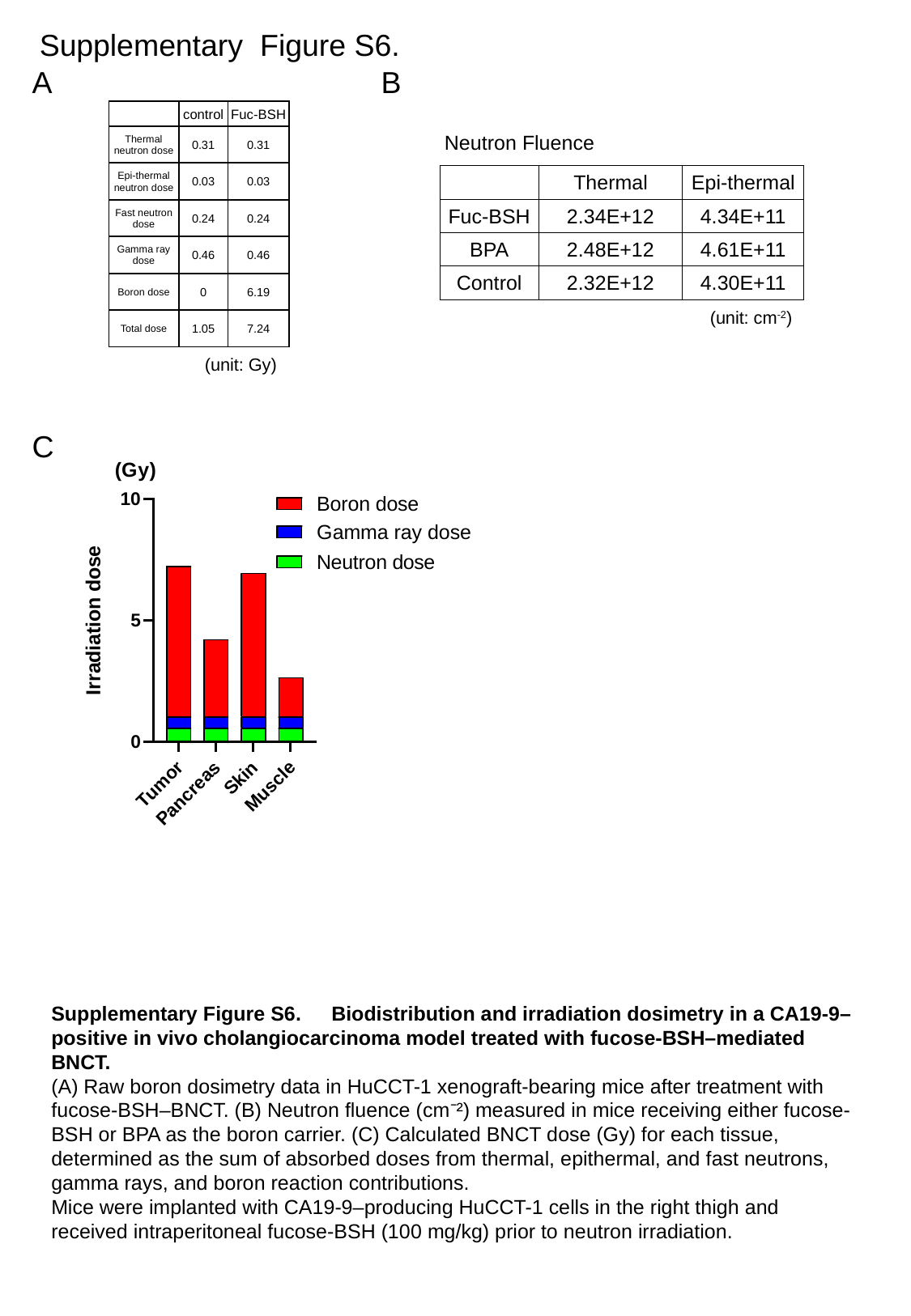

Supplementary Figure S6.
A B
C
| | control | Fuc-BSH |
| --- | --- | --- |
| Thermal neutron dose | 0.31 | 0.31 |
| Epi-thermal neutron dose | 0.03 | 0.03 |
| Fast neutron dose | 0.24 | 0.24 |
| Gamma ray dose | 0.46 | 0.46 |
| Boron dose | 0 | 6.19 |
| Total dose | 1.05 | 7.24 |
Neutron Fluence
| | Thermal | Epi-thermal |
| --- | --- | --- |
| Fuc-BSH | 2.34E+12 | 4.34E+11 |
| BPA | 2.48E+12 | 4.61E+11 |
| Control | 2.32E+12 | 4.30E+11 |
(unit: cm-2)
(unit: Gy)
Supplementary Figure S6.　Biodistribution and irradiation dosimetry in a CA19-9–positive in vivo cholangiocarcinoma model treated with fucose-BSH–mediated BNCT.
(A) Raw boron dosimetry data in HuCCT-1 xenograft-bearing mice after treatment with fucose-BSH–BNCT. (B) Neutron fluence (cm⁻²) measured in mice receiving either fucose-BSH or BPA as the boron carrier. (C) Calculated BNCT dose (Gy) for each tissue, determined as the sum of absorbed doses from thermal, epithermal, and fast neutrons, gamma rays, and boron reaction contributions.
Mice were implanted with CA19-9–producing HuCCT-1 cells in the right thigh and received intraperitoneal fucose-BSH (100 mg/kg) prior to neutron irradiation.
